# A long-acting octreotide analog formed by conjugation with a peptide linker modified with two fatty acids

**DOI:** 10.3389/fendo.2026.1871977

**Published:** 2026-08-25

**Authors:** Yueh-Hsiang Yu, Ya-Shan Chuang, Jessica Gehlert, Chi-Jiun Peng, Wei-Chen Lin, Mun-Teng Wong, Jon Wright, Hsing-Mao Chu, Carmay Lim, Tse Wen Chang

**Affiliations:** 1 T-E Meds, Inc., Taipei, Taiwan; 2 Immunwork, Inc., Taipei, Taiwan; 3 CMAX Clinical Research, Adelaide, SA, Australia

**Keywords:** acromegaly, albumin binding, IGF-1, neuroendocrine tumors, pharmacokinetics/pharmacodynamics, somatostatin analog

## Abstract

**Introduction:**

Current long-acting somatostatin analogs control acromegaly and neuroendocrine tumor symptoms, but rely on depot formulations requiring large-bore needle administration, often associated with injection-site pain, nodules, and variable drug release. To address this limitation, we developed TE-8214, a depot-free octreotide analog conjugated to a soluble, dual-fatty-acid albumin-binding module designed to enable prolonged systemic exposure while maintaining a fully aqueous formulation suitable for subcutaneous administration through a fine 30-gauge needle.

**Methods:**

TE-8214’s binding to human serum albumin (HSA) and somatostatin receptor 2 (SSTR2), and its receptor-mediated functional activity, were characterized in vitro and compared with octreotide. Pharmacokinetics and pharmacodynamics — suppression of serotonin, growth hormone, and insulin-like growth factor-1 (IGF-1) — were assessed in rodents and dogs following single or repeated dosing of TE-8214 or equimolar octreotide. Safety, tolerability, and pharmacokinetics of TE-8214 were then evaluated in a randomized, double-blind, placebo-controlled Phase 1 single-ascending-dose study in healthy adults (0.6–4.0 mg), with IGF-1 assessed as an exploratory pharmacodynamic biomarker.

**Results:**

In vitro, TE-8214 bound HSA while retaining potent SSTR2-mediated activity comparable to octreotide. In rodents and dogs, TE-8214's albumin binding extended its terminal half-life and enabled sustained suppression of serotonin, growth hormone, and IGF-1, exceeding the duration achieved with equimolar octreotide. In the Phase 1 study, TE-8214 was well-tolerated across all doses tested, with no serious or Grade ≥3 treatment-emergent adverse events, and produced sustained, dose-dependent reductions in IGF-1 consistent with systemic exposure; at the highest dose, IGF-1 fell by 22.5% from baseline and remained suppressed through Day 28.

**Discussion:**

These findings demonstrate that a depot-free, aqueous-formulated octreotide analog can achieve prolonged systemic exposure and sustained pharmacodynamic activity following a single dose in healthy volunteers. Although this study was not designed to evaluate therapeutic efficacy, the pharmacokinetic and pharmacodynamic profile of TE-8214 supports further clinical evaluation in patients with neuroendocrine tumors or acromegaly.

**Clinical trial registration:**

## Graphical Abstract

**Figure:**
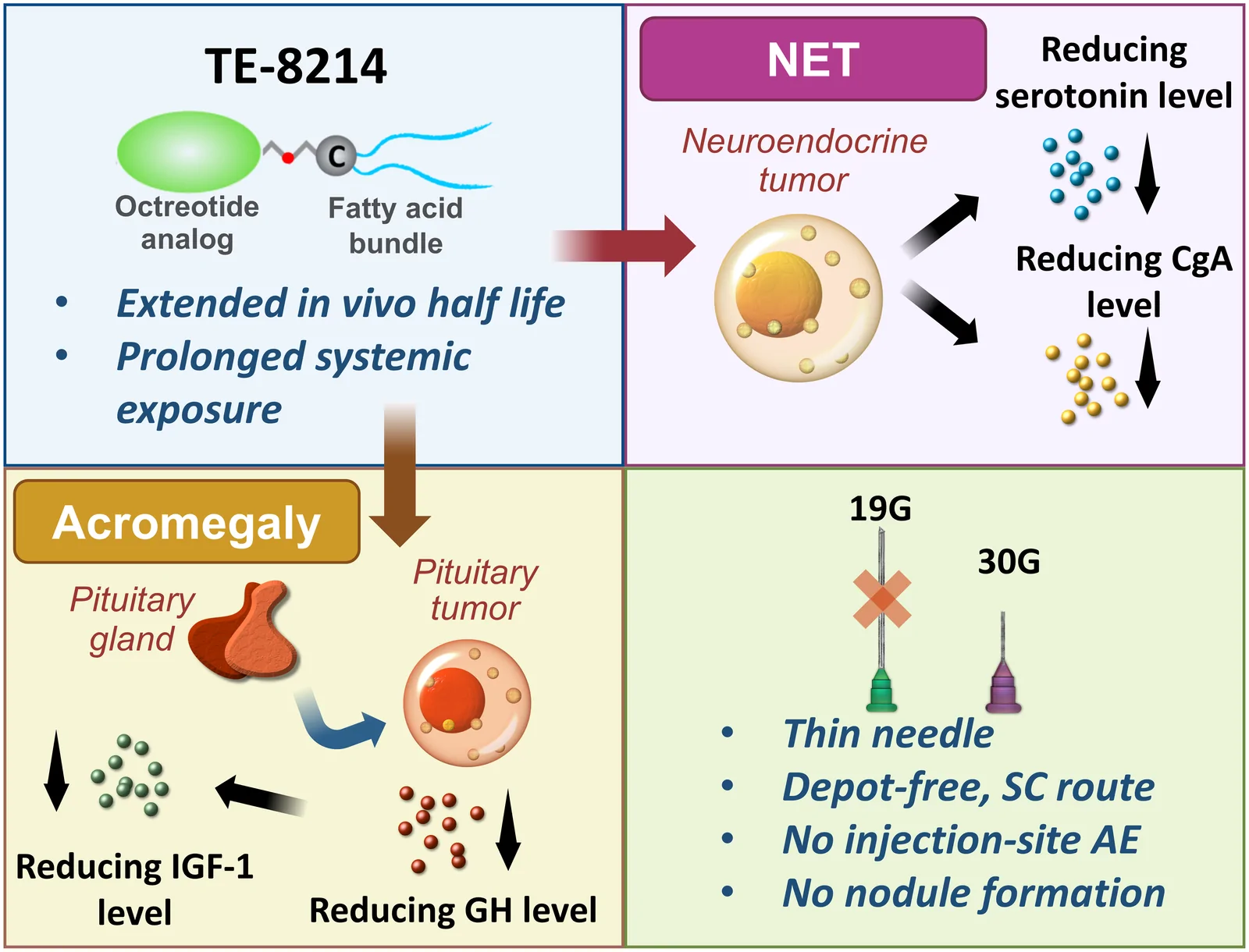
Table of Contents tumer graphic.

## Highlights

TE-8214 is the first depot-free, aqueous dual-fattyacid-conjugated octreotide analog to show a human terminal half-life of ~5.5 days and a statistically significant reduction in IGF-1 persisting to Day 28 after a single subcutaneous injection given through a fine 30-gauge needle without a depot formulation.

## 1 Introduction

Synthetic somatostatin analogs (SSAs) are cornerstone therapies for neuroendocrine tumors (NETs) and acromegaly, conditions driven by hormone hypersecretion from tumors overexpressing somatostatin receptors (SSTRs), particularly SSTR2 (1–5). Although native somatostatin effectively suppresses hormone release (6), its 1−3 minute plasma half-life (7) precludes therapeutic use. First-generation SSAs such as octreotide (Sandostatin^®^) extended the plasma half-life to ~100 minutes (8, 9), but require multiple daily subcutaneous (SC) injections, imposing a significant treatment burden that undermines patient adherence (10). Subsequent long-acting depot formulations (octreotide LAR, lanreotide Autogel, pasireotide LAR, or Oczyesa^®^) permit monthly dosing, but require 18−22 gauge needle injections, often causing injection-site pain, nodules, and/or inflammation (2, 11). Nodules can be mistaken for metastatic lesions during imaging, provoking patient anxiety (12), while repeated injections may cause localized induration and tissue hardening that complicate future administrations and create variable drug release (13, 14), potentially limiting efficacy. Collectively, these injection-site reactions pose adherence challenges to patients undergoing long-term therapy, highlighting a need for a long-acting SSA that maintains efficacy without the associated pain and administration burden.

**Figure 1:**
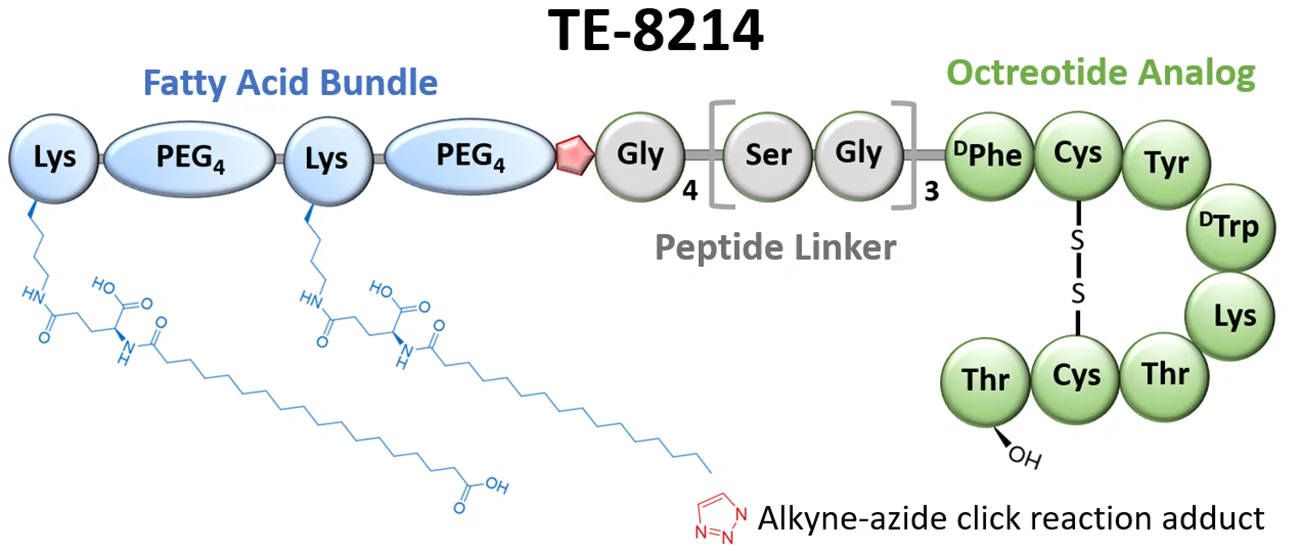
Schematic of TE-8214 comprising an octreotide analog (green) linked via an alkyne-azide click reaction adduct (pink) to a soluble 2FA bundle (blue) through a flexible peptide linker (gray) to preserve receptor binding or structural integrity. The heterologous C16-CH_3_/C18-COO^−^ (2FA) module enables high-affinity, reversible binding to HSA.

To address this unmet need, we developed TE-8214, a fully aqueous, depot-free dual-fatty-acid (2FA)-conjugated octreotide analog. The rational molecular design integrates three synergistic elements to preserve receptor activity, achieve sustained systemic exposure, and enable an aqueous formulation (**Figure 1**): (1) an octreotide-derived pharmacophore optimized for somatostatin receptor binding, (2) a heterologous 2FA bundle for high-affinity binding to human serum albumin (HSA), and (3) a hydrophilic, flexible (PEG_4_Lys)_2_ multi-arm linker (15, 16). We replaced Phe3 in octreotide by Tyr; this modification, clinically validated in the radiotracer DOTATOC, enhances SSTR2 selectivity, maintains biological potency, and enables radioiodination for pharmacokinetic tracking. While lipidated peptide therapeutics such as semaglutide (17) and tirzepatide (18) employ a single FA, TE-8214 incorporates two distinct FAs, C16-CH_3_ and C18-COO^−^, attached to lysine residues on a solubilizing (PEG_4_Lys)_2_ linker. This heterologous 2FA configuration was designed to provide complementary geometric and electrostatic environments with albumin’s multiple FA-binding pockets, thereby enhancing affinity for HSA whose half-life is ~19 days (19). Albumin binding increases TE-8214’s apparent molecular size and is expected to reduce renal filtration and proteolytic degradation, creating a circulating reservoir that prolongs systemic exposure (15). The hydrophilic (PEG_4_Lys)_2_ linker was designed to offset the increased hydrophobicity associated with dual lipidation, enabling a low-viscosity solution suitable for SC injection via a fine 30-gauge needle. By combining 2FA–mediated albumin binding with a solubilizing linker and a clinically validated octreotide analog scaffold, this molecular design strategy can achieve sustained systemic exposure without reliance on depot-based delivery systems.

This work comprehensively validates our strategy, from molecular design to first-in-human evaluation of TE-8214, focusing on safety, tolerability and pharmacokinetics. Consistent with early-phase clinical development principles, the study was conducted in healthy volunteers to establish translational exposure–response relationships prior to patient-based efficacy studies. We demonstrate that TE-8214 binds both HSA and SSTR2, retaining potent octreotide-like function *in vitro*. Preclinically, TE-8214 exhibits a markedly extended terminal half-life and outlasts equimolar octreotide for hormone control across rodents and canines. In a randomized, double-blind, placebo-controlled Phase 1 single-ascending dose study in healthy adults, TE-8214 was well-tolerated at single doses of 0.6–4.0 mg. A SC 4.0 mg dose achieved a terminal half-life of 5.5 days and sustained suppression of insulin-like growth factor-1 (IGF-1), a key disease biomarker (20, 21) through Day 28. Together, these findings demonstrate that a depot-free, aqueous-formulated SSA can achieve multi-day human pharmacokinetics and 28-day pharmacodynamic suppression from a single fine-needle SC injection in healthy volunteers, providing translational support for subsequent efficacy studies in patients.

## 2 Materials and methods

### 2.1 TE-8214 synthesis and characterization

The 2FA-conjugated octreotide analog, TE-8214, was synthesized by SynTheAll Pharmaceutical Co., Ltd (Jiangsu, China) under Good Manufacturing Practice (GMP) standards using qualified, GMP-grade reagents. The synthesis proceeded in three main stages (**Supplementary Figures 1**, **2**).

#### 2.1.1 Step 1 — synthesis of azide-modified octreotide peptide (compound 1)

The linear octreotide analog backbone was assembled on 2-chlorotrityl chloride (2-CTC) resin via standard Fmoc/tBu/Boc solid-phase peptide synthesis (SPPS), starting from Fmoc-L-Thr(tBu)-OL as the C-terminal residue (**Supplementary Figure 1**). Chain elongation proceeded through repeated cycles of (i) Fmoc deprotection using 20% piperidine in DMF (20–30 °C, 25–35 minutes) and (ii) amino acid coupling. The incoming Fmoc-amino acid was pre-activated with Oxyma and DIC (or HATU and DIEA for Fmoc-Gly-Gly-OH) in DMF or NMP at 15–25 °C, then transferred to the resin and agitated at 20–30 °C until completion, as judged by Kaiser test. To enable the subsequent click reaction with the 2FA-bundle, 2-azidoacetic acid was coupled to the N-terminus. The peptide was cleaved from the resin, deprotected using 95% trifluoroacetic acid, 2.5% triisopropylsilane, 2.5% H_2_O at 20–30 °C for 90–150 minutes, and precipitated with cold methyl tert-butyl ether. The intramolecular disulfide bond was formed by oxidation with 0.05 M I_2_ in methanol at 20–30 °C for 10–25 minutes and the reaction was quenched with 0.05 M sodium thiosulfate in water at 20–30 °C for 5–15 minutes. Compound **1** was purified by preparative reverse-phase high-performance liquid chromatography (HPLC) (YMC-Triart Prep C18-S, 12nm, 10μm) using a gradient of acetonitrile in 50 mM triethylammonium phosphate (TEAP) buffer, pH 2.6.

#### 2.1.2 Step 2 — synthesis of alkyne-functionalized 2FA-bundle (compound 2)

The 2FA-linker module was similarly assembled on 2-CTC resin by SPPS, as depicted in **Supplementary Figure 2**. The resin was first loaded with ivDde-L-Lys(Fmoc)-OH. Orthogonal protection strategies utilizing Fmoc (deprotected via 20% piperidine/DMF) and ivDde (deprotected via 5% hydrazine/DMF) allowed for the site-specific, stepwise coupling of amino acids, FAs, PEG_4_ spacers, and the alkyne-acid cap using HATU and DIEA in DMF. Specifically, Fmoc removal exposed the lysine ϵ-amine, which was coupled sequentially with Fmoc-Glu-OtBu and octadecanedioic acid, 1-(1,1-dimethylethyl) ester (C18-COO^−^ FA), both activated with HATU and DIEA in DMF at 20–30 °C. The ivDde protecting group was then removed using 5% hydrazine in DMF to enable coupling of Fmoc-NH-(PEG)_4_-propionic acid using HATU and DIEA in DMF. After Fmoc removal, a second branching lysine (Fmoc-L-Lys(ivDde)-OH) was introduced via HATU/DIEA coupling, providing two orthogonally protected amines for independent elaboration. The amino group of the second lysine (after Fmoc removal) was extended with a second Fmoc-NH-(PEG)_4_-propionic acid spacer and capped with 4-pentynoic acid to install the terminal alkyne required for copper-catalyzed azide-alkyne cycloaddition (CuAAC) conjugation. The ivDde group on the NH group of the second lysine was then removed with 5% hydrazine in DMF, and the exposed amine was sequentially coupled with Fmoc-Glu-OtBu and palmitic acid (C16-CH_3_ FA). The final alkyne-functionalized 2FA-linker module (compound **2**) was cleaved from the resin, globally deprotected, and purified by preparative reverse-phase HPLC (YMC-Triart Prep C18-S 12nm S-10μm, acetonitrile/20 mM NH_4_HCO_3_, gradient), and characterized by LC-MS to confirm the identity of compound **2**.

#### 2.1.3 Step 3 — CuAAC conjugation to yield TE-8214 (compound 3)

The azide-modified octreotide (1.0 eq) and the alkyne-functionalized 2FA-linker (1.3 eq) were conjugated via CuAAC in a 1:1 water/acetonitrile mixture containing CuI (2.8 eq) and DIEA (22.3 eq) at 35 °C for 30 minutes (**Supplementary Figure 1**). Reaction progress was monitored by analytical reverse-phase HPLC, quenched with EDTA (3.5 eq), and diluted with 50% acetonitrile in water (v/v).

#### 2.1.4 Purification and characterization of TE-8214

Compound **3** was purified by preparative reverse-phase HPLC on a C18 column (UniSil 10–120 C18, 21.2 × 250 mm) using a linear gradient of acetonitrile (30–40%) in 50 mM NaHCO_3_ (aq.) over 40 minutes at a flow rate of 20 mL/min, with UV detection at 220 and 280 nm. Purity (>95%) was verified by analytical ultra-performance liquid chromatography (UPLC) using a Waters Acquity CSH Phenyl-Hexyl column (2.1 × 150 mm, 1.7 μm) at 60 °C, with a gradient of 0.1% TFA in water (A) and 0.1% TFA in acetonitrile (B) from 10% to 95% B over 30 min at a flow rate of 0.4 mL/min, monitored at 214 nm. Molecular identity was verified by LC–MS, which showed an observed mass of 3418.71 Da (calculated: 3418.75 Da).

#### 2.1.5 Drug product formulation

The pharmacokinetics and toxicology studies described below employed an aqueous solution comprising 6 mg mL^-1^ TE-8214, 1.42 mg mL^-1^ disodium phosphate dihydrate, 14.0 mg mL^-1^ 1,2-propanediol, 9.0 mg mL^-1^ benzyl alcohol, 0.1 mg mL^-1^ Tween 20, and 1.0 mg mL^-1^ Tween 80.

### 2.2 *In vitro* studies

HSA used in all *in vitro* binding studies was FA-free and globulin-free so that binding of TE-8214 to the FA-binding sites on albumin could be measured without competition from endogenous lipids.

#### 2.2.1 Surface plasmon resonance binding assays

Binding to HSA and SSTR2 was measured on a Biacore T200 system (GE Healthcare). 2 μg mL^-1^ HSA (A3782, Sigma-Aldrich) or SSTR2 (HTS028M, Merck) was immobilized on a Biacore CM5 sensor chip (~880 RU). Analyte solutions containing TE-8214 (0.3125–40 μM) were flowed over the chip at a rate of 30 μL min^-1^ at 25 °C in a running buffer (pH 7.4) containing phosphate-buffered saline (PBS) with 5% DMSO (9224-03, J.T.Baker) for HSA (association 30s, dissociation 180s), or 2% DMSO and 0.5% surfactant P20 (BR100054, Cytiva) for SSTR2 (association 40s, dissociation 240s). The chip surface was regenerated with 50 mM NaOH for 30 seconds between cycles. Sensorgrams for each target (HSA or SSTR2) were fitted to a 1:1 Langmuir binding model using Biacore T200 evaluation software to derive K_D_ values.

#### 2.2.2 Cell lines and culture

Human pancreatic neuroendocrine tumor cell line (QGP-1, JCRB0183) was purchased from Japanese Collection of Research Bioresources Cell Bank. Human malignant meningioma (IOMM-Lee, ATCC CRL-3370), rat pancreatic tumor (AR42J, ATCC CRL-1492), and rat pituitary tumor (GH3, ATCC CCL-82.1) cell lines were purchased from American Type Culture Collection (ATCC). QGP-1 cells were cultured in RPMI 1640 (Gibco), IOMM-Lee in DMEM (Gibco), and AR42J or GH3 in F-12K medium, each supplemented with 10% fetal bovine serum (Gibco) and antibiotic-antimycotic solution (Corning). Cultures were maintained at 37 °C in 5% (v/v) CO_2_ humidified incubator and confirmed to be free of mycoplasma contamination using a PCR mycoplasma detection kit (Biosmart).

#### 2.2.3 Functional cellular assays

##### 2.2.3.1 Cyclic adenosine monophosphate reduction

QGP-1 or AR42J cells (1 × 10^5^ per well, 48-well plate) were cultured overnight and incubated with various concentrations of TE-8214 or octreotide for 20 minutes at 37 °C. After removing the treatment solution, cells were lysed with 0.1 M HCl, centrifuged, and cell lysates were diluted with ELISA buffer and analyzed for cAMP using a Correlate-EIA Direct Assay kit (581001, Cayman). A four-parameter standard curve was generated using Gen5 to calculate the cAMP concentrations.

##### 2.2.3.2 Serotonin/chromogranin A secretion

QGP-1 and AR42J cells (2 × 10^4^ per well, 96-well plate) were cultured overnight, rinsed with PBS, and incubated with various concentrations of TE-8214 or octreotide for two days. For QGP-1 cells, the culture medium was collected and centrifuged to obtain the supernatant, whereas AR42J cells were lysed with RIPA buffer and cell lysates were collected after centrifugation. Extracellular serotonin in QGP-1 supernatants (diluted 1:5 with assay buffer) was quantified by ELISA (ADI-900-175, Enzo) by measuring optical density at 405 nm using a Synergy H1 multi-mode microplate reader. Intracellular CgA levels in AR42J cell lysates (diluted 1:5 with Standard/Sample Diluent) was quantified by Rat CgA ELISA (RK03567, ABclonal) by measuring optical density at 450 nm. A four-parameter regression standard curve (generated in Gen5) corrected for the 5-fold dilution was used to calculate the serotonin/CgA concentration in each sample.

##### 2.2.3.3 Cell viability

QGP-1, IOMM-Lee, AR42J, or GH3 cells (5 × 10^3^ per well, 96-well plate) were separately seeded in 50 µL medium and after 16–18 hours, incubated with 50 µL TE-8214 or octreotide for 3 days, followed by 10 µL of alamarBlue™ Cell Viability Reagent (Invitrogen) for 1–5 hours to assess cell viability. Fluorescence was measured hourly using a Synergy H1 multi-mode microplate reader with 560 nm excitation wavelength and 590 nm emission wavelength.

For all functional assays, dose-response data were fitted to a four-parameter variable slope model in GraphPad Prism to compute EC_50_ values.

### 2.3 *In vivo* studies

#### 2.3.1 Animals

All animal studies complied with internationally accepted ethical guidelines for laboratory animal care and use and were approved by the Institutional Animal Care and Use Committee (IACUC: IACUC-18–209 and NLAC(TN)-109-M-014). All rodent experimental protocols were reviewed and agreed upon by the IACUC Committee of the National Defense Medical Center (IACUC-18-209). All dog procedures were approved by the IACUC (NLAC(TN)-109-M-014) of Taiwan’s National Center for Biomodels (NCB).

##### 2.3.1.1 Rodents

Male Sprague-Dawley (SD) rats (8 weeks old) and male/female NOD.CB17-Prkdcscid/NcrCrlBltw mice (6 weeks old) were purchased from Taiwan’s National Center for Biomodels (NCB) and the Animal Facility at Academia Sinica, Taiwan, respectively. The study was performed according to the Standard Operating Procedures (SOPs) of the National Defense Medical Center. Rats were housed 2–3 per cage and mice 3–5 per cage, in well-ventilated, humidified-controlled rooms (14 h light/10 h dark cycle) and were fed standard chow with unrestricted water access. Prior to experiments, mice were accustomed to blood sampling and dosing procedures for a week to minimize handling stress. Rats were anesthetized using isoflurane (Matrx™ VIP 3000 Calibrated Vaporizer, Midmark). Induction was performed in a pre-filled chamber with 3–4% (v/v) isoflurane for 3–5 minutes until the loss of the righting reflex was confirmed. To maintain the anesthetic state during procedures, rats were fitted with a customized inhalation mask delivering a continuous supply of isoflurane. At the end of the study, euthanasia was performed in strict accordance with the AVMA Guidelines for the Euthanasia of Animals. Euthanasia was conducted via carbon dioxide (CO_2_) inhalation, with death confirmed by cervical dislocation. All procedures were approved by the IACUC.

##### 2.3.1.2 Dogs

Three male non-naïve beagle dogs (3 years old) were acquired from Oriental Yeast Co., Ltd. (Tokyo, Japan). The study was conducted by NCB, excluding the sample analysis. After a two-week acclimation with daily clinical monitoring, dogs were group-housed in an environmentally controlled room (19−25 °C, 30−70% relative humidity, 12 h light/dark cycle) with enrichment (music, toys, exercise). The dogs were fed 300 g of canine diet and had unrestricted access to filtered reverse osmosis water that met NCB’s quality standards. At study completion, dogs were examined by a veterinarian and placed in an adoption program. All procedures in beagle dogs were performed in conscious animals without anesthesia. To minimize stress and ensure stable physiological conditions, animals were acclimated to the handling procedures and gently restrained by trained technicians during subcutaneous administration and blood collection via the jugular vein. The disposition of animals varied based on the study site and protocol. For studies conducted at NCB, the procedures were non-terminal; following the completion of the experiments, all animals were clinically evaluated and successfully released for adoption in accordance with institutional animal welfare policies. For the toxicology studies conducted at the CRO, euthanasia was performed in strict accordance with the AVMA Guidelines for the Euthanasia of Animals. Animals were sedated via an intramuscular or intravenous injection of 5 mg/kg Zoletil (or 5–10 mg/kg ketamine), followed by an intravenous injection of 80 mg/kg xylazine hydrochloride. Death was then induced via exsanguination through the femoral artery to facilitate necropsy.

#### 2.3.2 Protein-binding studies

Plasma protein binding of TE-8214 was assessed using plasma from CD-1 mouse, SD rat, beagle dog, cynomolgus monkey (Medicilon Preclinical Research, Shanghai), and human (ZenBio, Research Triangle Park, NC). TE-8214 (0.5, 5, or 50 μM) in 100 μL plasma was dialyzed against 100 μL PBS in a high-throughput equilibrium dialysis device with pre-treated membrane strips (Apricot Designs) for 5 hours at 37 °C with orbital shaking (150 rpm). Post-incubation, plasma chamber samples (2.5 μL diluted to 25 μL with blank plasma + 25 μL PBS) and buffer chamber samples (25 μL + 25 μL blank plasma) were analyzed by liquid chromatography-tandem mass spectrometry (LC-MS/MS). Percent bound was calculated as ([plasma] – [buffer])/[plasma] × 100, where [plasma] and [buffer] are TE-8214 concentrations in plasma and PBS chambers, respectively. The validated LC-MS/MS assay had a lower limit of quantification (LLOQ) of 1 ng/mL. Samples with free-drug concentrations below the LLOQ were reported as below quantification; therefore, the reported protein binding represents the minimum estimated bound fraction.

#### 2.3.3 Pharmacokinetic studies

##### 2.3.3.1 SD rats

To determine the concentration of TE-8214 in SD rats, TE-8214 was radiolabeled with iodine-131 using the modified chloramine-T method. ^131^I-TE-8214 was purified by centrifugal filtration (UFC5003, Merck) to remove free iodine and its radiochemical purity (>98%) was confirmed by reverse-phase thin-layer chromatography (1.05559.0001, Merck). After mixing purified ^131^I-TE-8214 with 0.9% saline, male rats (n = 3) received single SC (124.5 μg kg^-1^; injection volume 0.2 mL) or intravenous (IV) (102.6 μg kg^-1^; injection volume 0.1 mL) doses of ^131^I-TE-8214 for 4 days. TE-8214 concentrations were determined by measuring radioactivity in blood samples collected up to 96 h post-dose (SC: 1, 2, 4, 17.5, 24, 48, 72, 96 h; IV: 0.5, 1, 2, 4, 24, 27, 48, 51, 72, 96 h). At each time point, 60 μL of blood was transferred to a polypropylene gamma tube (6005230, PerkinElmer) and radioactivity measured by a gamma counter (2470 WIZARD^2^, PerkinElmer), expressed as counts per minute (cpm). The total drug concentration in each blood sample was determined according to Equation 1:

(1) [I131-TE-8214]=[radioactivity(cpm mL−1)/theoretical injected radioactivity]×dose

##### 2.3.3.2 Dogs

Male dogs (n = 3) received a single SC dose of TE-8214 (40 µg kg^-1^; injection volume 0.04 mL kg^-1^) over 29 days. Plasma samples were collected at 0 h (pre-dose), 2 h, 4 h, and 6 h (Day 0), and on Days 1, 2, 3, 6, 9, 13, 15, 17, 21, 24, and 28. Blood was collected in heparinized tubes (BD Vacutainer^®^), centrifuged at 1,500 × g for 10 mins at 2–8 °C for harvesting plasma. The concentration of TE-8214 in plasma was detected by LC-MS/MS. Briefly, 50 µL of standard, quality control, blank plasma, or plasma sample was mixed with 150 µL methanol, vortexed, and centrifuged at 20,000 × g for 10 min. Supernatant (50 µL) was transferred, and 10 µL injected for analysis by LC-MS/MS using a ZORBAX RR Eclipse Plus C18 column (95 Å, 4.6 × 50 mm, 3.5 µm) with a mobile phase of water (0.1% formic acid, A) and acetonitrile (0.1% formic acid, B) at a flow rate of 0.8 mL min^-1^. A gradient elution was applied: 60% A/40% B (0–0.1 min) to 5% A/95% B (1–1.4 min), returning to 60% A/40% B (1.5–2.5 min). MS detection was performed in positive electrospray ionization mode, using multiple reaction monitoring of the transition m/z 1141.4 → 819.5 for TE-8214. The instrument parameters were: declustering potential 100 V, entrance potential 10 V, collision energy 45 V, collision cell exit potential 30 V; ion spray voltage 5,000 V; source temperature 550 °C; curtain gas 40 psi; collision gas high; and nebulizing (GS1) 50 nitrogen at psi; auxiliary (GS2) nitrogen at 60 psi. The blood and plasma concentrations of TE-8214 were determined using a 4-parameter logistic regression model by Prism software.

Pharmacokinetic parameters for both species were calculated using non-compartmental analysis with PKSolver software.

#### 2.3.4 Single-dose efficacy in QGP-1 CDX mice

To evaluate the efficacy of TE-8214 in reducing serotonin levels, a neuroendocrine CDX mouse model was established using the SSTR-expressing QGP-1 neuroendocrine tumor cell line. To generate SC tumors, 5 × 10^6^ QGP-1 cells in PBS, along with 50% (v/v) Matrigel solution (354262, Corning), were subcutaneously injected into the flanks of NOD-SCID mice. Twenty-four days post-inoculation, when the mean tumor volume was 350 ± 100 mm^3^, six QGP-1 CDX mice (3 males, 3 females) received a single SC dose of TE-8214 (0.34 mg kg^-1^) or equimolar octreotide (0.11 mg kg^-1^), each delivered in an injection volume of 0.1 mL. Approximately 0.02 mL of blood was collected via the retro-orbital route. Plasma was collected by centrifugation at 1,500 × g for 10 minutes at 4 °C. Plasma serotonin levels were measured over 7 days at Day 24 (pre-dose and 6 hr post-dose), and Days 25, 28, and 31, using a Serotonin ELISA kit (ADI-900175, Enzo).

#### 2.3.5 Single-dose efficacy in SD rats

To assess the ability of TE-8214 to reduce IGF-1 levels, male SD rats received a single SC dose of TE-8214 (13, 66, 100, 200, or 330 µg kg^-1^), 100 µg kg^-1^ octreotide (APExBio) or PBS (negative control), each administered in an injection volume of 0.3 mL kg^-1^. 7–10 plasma samples (≈0.2 mL blood each) were obtained from the tail vein of rats. Plasma was isolated by centrifugation (1,500 × g, 10 minutes, 4 °C) and diluted with PBS. Plasma IGF-1 concentration was determined with a commercial Rat IGF-1 ELISA kit (MBS2700908, MyBioSource). 100 µL of diluted plasma or standards were applied to a 96-well pre-coated plate, and optical density was measured at 450 nm using a Synergy H1 multi-mode plate reader. A four-parameter logistic regression standard curve (generated in Gen5), corrected for dilution factors, was used to calculate the IGF-1 concentration in each sample.

#### 2.3.6 Single-dose efficacy in beagle dogs

To assess the pharmacodynamic effect of TE-8214 on plasma IGF-1 and growth hormone (GH), three male beagle dogs (3 years old, 12–14 kg) received a single SC dose of TE-8214 (40 µg kg^-1^; injection volume 40 µL kg^-1^). Fifteen blood samples (~0.7 mL each) were collected from the cephalic or saphenous vein at 0, 2, 4, and 6 hr on Day 0, and on Days 1, 2, 3, 6, 9, 13, 15, 17, 21, 24, and 28 post-dose. Blood was collected into heparinized tubes and centrifuged at 1,500 × g for 10 minutes at 2–8 °C to harvest plasma. Plasma GH concentrations were determined using a canine GH ELISA kit (MBS287193, MyBioSource), and IGF-1 concentrations with a high-sensitivity IGF-1 ELISA kit (MBS2707964, MyBioSource). Both GH and IGF-1 concentrations were measured according to the kit instructions and their analyses followed protocols described for the rat single-dose study.

#### 2.3.7 Multiple-dose efficacy in SD rats

To evaluate the effect of repeated dosing, male SD rats (n = 4/group) received once-weekly SC injections of TE-8214 (50 or 100 µg kg^-1^; injection volume 0.1 mL per dose) for six weeks. 21 plasma samples (~0.2 mL blood each) were collected from the tail vein of rats at Day 0 (pre-dose), Day 1, Day 4, Day 7 (pre-dose), Day 8, Day 11, Day 14 (pre-dose), Day 15, Day 18, Day 21 (pre-dose), Day 22, Day 24, Day 28 (pre-dose), Day 29, Day 32, Day 35 (pre-dose), Day 36, Day 39, Day 42, Day 46, and Day 49. Plasma IGF-1 were measured using Rat IGF1 ELISA kit (MBS2700908, MyBioSource) and GH levels Rat with GH ELISA kit (MBS267670, MyBioSource). Plasma preparation and IGF-1 analysis followed the single-dose rat protocol. GH concentration was measured according to the kit instructions.

### 2.4 Dose toxicity studies

All repeated-dose toxicology and safety pharmacology studies were conducted at a GLP-compliant contract research organization (CRO) in accordance with the SOPs and the Guide for the Care and Use of Laboratory Animals. The study protocols were reviewed and approved by the IACUC of the testing facility.

#### 2.4.1 SD rats

A total of 160 SPF-grade SD rats (80M/80F) were utilized. 120 animals were assigned to the toxicity study (n = 30 per group, 15M/15F) and 40 to a satellite toxicokinetic study. The rats received twice-weekly SC injections of TE-8214 for 9 doses at 0 (vehicle), 2, 6, or 18 mg kg^−1^ (injection volume 3 mL kg^-1^ across all groups), followed by a 4-week recovery period. In-life assessments included clinical observations, local irritation scoring, body weight, and food consumption. Pharmacodynamic effects were monitored via IGF-1 levels. At the dosing and recovery endpoints, comprehensive clinical pathology, ophthalmoscopy, and histopathological examinations were conducted. Systemic exposure was characterized on Days 1 and 26 using a validated LC-MS/MS method.

#### 2.4.2 Dogs

Forty Beagle dogs (5–9 months old, 5M/5F per group) received weekly SC injections of TE-8214 for 5 doses at 0, 0.8, 2.5, or 7.0 mg kg^−1^ (injection volume 1.25 mL kg^-1^ across all groups), followed by a 4-week recovery period. Systemic toxicity was evaluated through clinical observations, ophthalmology, and clinical pathology. Cardiovascular and respiratory safety pharmacology was assessed via non-invasive monitoring. Systemic exposure and toxicokinetics were characterized using a validated LC-MS/MS method on Days 1 and 22. Comprehensive necropsy and histopathological examinations were performed at the end of the dosing and recovery phases to determine the No Observed Adverse Effect Level (NOAEL).

### 2.5 Phase 1 clinical trial (TE-8214-001)

#### 2.5.1 Study design and participants

This first-in-human, Phase 1, randomized, double-blind, placebo-controlled, single-ascending dose study was sponsored by Immunwork, Inc. and conducted by CRO, Novotech, at one site in Australia (ClinicalTrials.gov ID, NCT06372652) between June 18−December 6, 2024. Eligible participants were healthy adults (18–64 years old), confirmed by medical history, physical examination, vital signs, clinical laboratory tests, and electrocardiogram. Key exclusion criteria included clinically significant abnormalities in laboratory values, physical examination, or electrocardiogram; acute illness within 2 weeks prior to dosing; hypersensitivity to SSAs or related compounds; history of severe allergy (including anaphylaxis), pancreatic injury, pancreatitis, vitamin B12 deficiency, hypothyroidism (TSH >4 mIU/L), diabetes mellitus, or HbA1c ≥6.5% at screening. Investigators could exclude participants if any condition was judged likely to compromise safety, protocol compliance, or data interpretation.

#### 2.5.2 Randomization and masking

**Figure 2:**
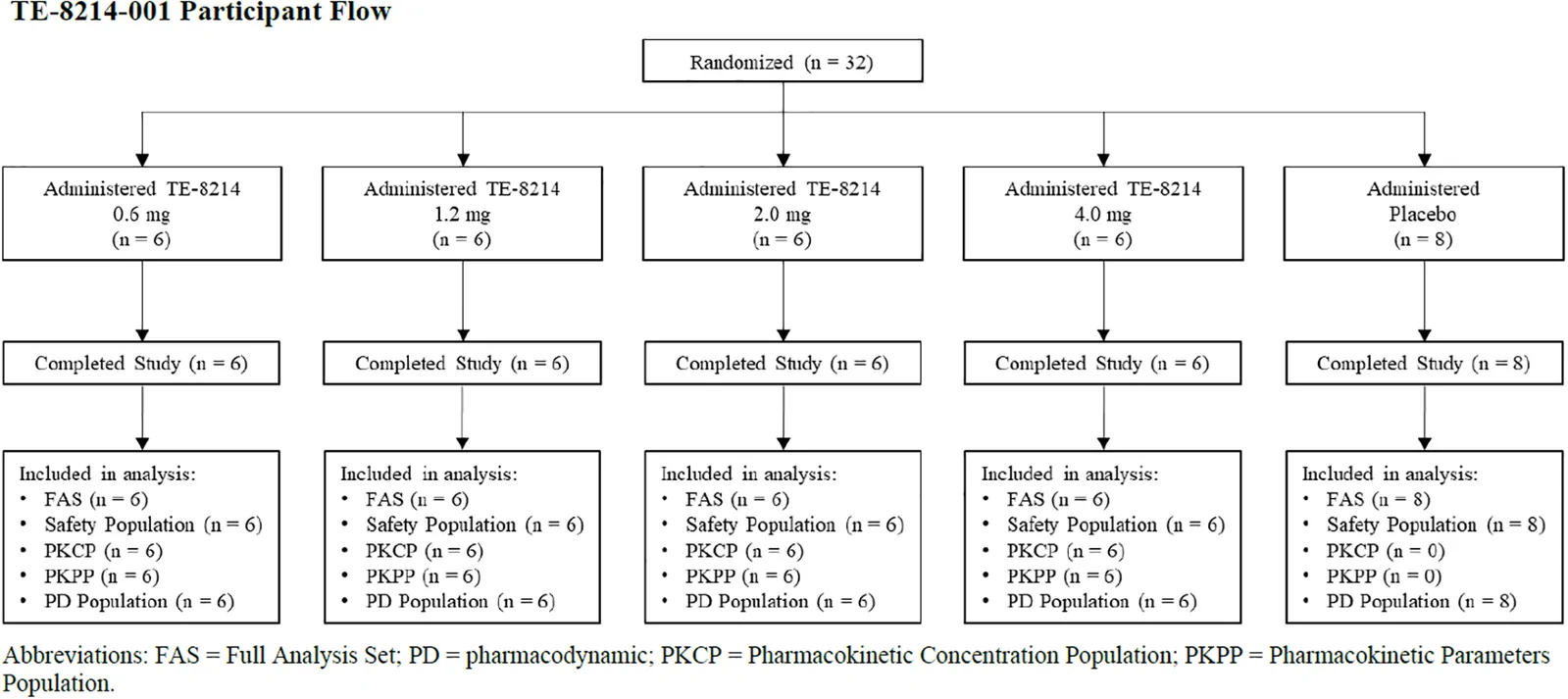
Participant flow and analysis populations in the TE-8214-001 Phase 1 clinical trial. Thirty-two participants were randomized into four sequential dose-ascending cohorts to receive a single subcutaneous dose of TE-8214 (0.6, 1.2, 2.0, or 4.0 mg; n = 6 per cohort) or placebo (n = 8). All 32 participants completed the study. The full analysis set (FAS), safety population, pharmacokinetic concentration population (PKCP), pharmacokinetic parameters population (PKPP), and pharmacodynamic population (PD Population) are shown for each treatment group.

Participants were randomized into 4 sequential dose-ascending cohorts to receive a single SC injection of TE-8214 (0.6, 1.2, 2.0, or 4.0 mg; n = 6 per cohort) or placebo (saline; n = 8 total), administered subcutaneously into the abdomen using a 30-gauge needle (see **Figure 2** below). Randomization was computer-generated, and both participants and investigators were blinded to treatment allocation. In each cohort, the first two participants (sentinel group) were randomized 1:1 to TE-8214 or placebo. If no treatment-related SAE or Grade ≥3 treatment-emergent adverse events (TEAE) occurred within 24 hours, dosing proceeded in the remaining six participants (randomized 5:1 to receive TE-8214 or placebo). Dose escalation to the next cohort required ≥14 days of blinded safety data from six participants in the preceding cohort, supplemented with available pharmacokinetics/pharmacodynamics data, and was approved by an independent Scientific Review Committee.

#### 2.5.3 Study procedures

The trial comprised three phases: (i) screening for eligibility (Day −28 to Day −1), (ii) randomized double-blind dosing (Day 1), and (iii) follow-up. The follow-up extended to Day 84 for Cohorts 1−2, and the first six participants in Cohort 3, and to Day 56 for the final two participants in Cohort 3 and all participants in Cohort 4. All 32 participants completed the study.

#### 2.5.4 Study endpoints

The primary endpoint was safety and tolerability of TE-8214, assessed by the incidence of TEAEs, changes in clinical laboratory values (hematology, coagulation, blood biochemistry, urinalysis), physical examinations, vital signs, body weight, and electrocardiograms. The secondary endpoint was to estimate pharmacokinetic parameters (*C_max_*, *T_max_*, *AUC*_0–*t*_, *AUC*_0–∞_, *t*½, *λ_z_*, *CL/F, V_z_/F*) based on TE-8214 plasma concentrations. The exploratory endpoint was to evaluate pharmacodynamics, assessed by changes from baseline in serum IGF-1 levels.

### 2.6 Ethics compliance

The repeated-dose toxicology and safety pharmacology studies were conducted at an independent, GLP-compliant CRO (Section 3.4). The Phase 1 clinical trial was conducted by an independent CRO (Novotech) under the oversight of a local institutional review board and an independent Scientific Review Committee that approved dose escalation (Section 3.5). The clinical study was conducted in accordance with the Declaration of Helsinki, the Council for International Organizations of Medical Sciences International Ethical Guidelines, and the International Council for Harmonisation of Technical Requirements for Pharmaceuticals for Human Use - Good Clinical Practices Guideline. All participants provided written informed consent prior to enrollment.

### 2.7 Statistical analysis

#### 2.7.1 Non-clinical studies

Preclinical studies were exploratory hypothesis-generating pharmacology studies using small cohorts (n = 3–4) in accordance with the principles of Replacement, Reduction and Refinement. These studies were neither randomized nor blinded, and no formal power calculations were performed. However, the GLP toxicology studies employed larger cohorts with a total of 160 SD rats (80M/80F) and 40 beagle dogs (20M/20F). Data were analyzed using GraphPad Prism version 8.0 (GraphPad Software, La Jolla, CA) and are presented as mean ± SD. Comparisons between two groups used unpaired two-tailed Student’s t-test with p < 0.05 considered significant. EC_50_ values were estimated by nonlinear regression using a four-parameter variable-slope model.

#### 2.7.2 Clinical study

The safety population included all randomized participants who received a TE-8214 dose. Adverse events were coded using the Medical Dictionary for Regulatory Activities (MedDRA^®^ version 27.0). Pharmacokinetic parameters were calculated by non-compartmental analysis using Phoenix WinNonlin^®^ software (version 8.4 or higher, Certara, Radnor, PA, USA). Percentage changes from baseline in IGF-1 were analyzed using mixed model two-way ANOVA with Dunnett’s correction for multiplicity.

## 3 Results

### 3.1 TE-8214 design and synthesis using a modular 2FA platform

We generated TE-8214 using a modular 2FA platform, detailed in our previous work (15). This platform employs a soluble (PEG_4_Lys)_2_ linker to precisely position two distinct FAs, creating an albumin-binding 2FA bundle. Among FA combinations tested, the C16-CH_3_/C18-COO^−^ hybrid produced greater and/or more durable serotonin/IGF-1 reductions in NOD SCID mice with QGP-1 tumors and SD rats than two C16 monoacids and/or two C18/C20 diacids (**Supplementary Figure 3**). Hence, this hybrid was selected for developing TE-8214.

First, an octreotide analog incorporating an azide-functionalized residue was prepared by SPPS (**Supplementary Figure 1**, compound **1**). Next, a 2FA bundle, consisting of a (PEG_4_Lys)_2_ core bearing the C16-CH_3_/C18-COO^−^ hybrid with a N-terminal alkyne group, was synthesized (**Supplementary Figure 2**, compound **2**). It was then site-specifically conjugated to the octreotide analog using CuAAC, chosen for its high efficiency, specificity, and biocompatibility. The final conjugate, TE-8214, was purified by reverse-phase HPLC to >98% purity, as confirmed by analytical UPLC (**Supplementary Figure 4**). Its molecular identity was confirmed by liquid chromatography-mass spectrometry (LC-MS), which gave an observed molecular weight of 3418.71 Da (**Supplementary Figure 5**), closely matching the theoretical value of 3418.75 Da. TE-8214 (≤6 mg mL^-1^) remained stable after 24 months storage at 5 ± 3 °C, as shown by the UPLC-UV and SEC-HPLC chromatograms (**Supplementary Figure 6**).

### 3.2 TE-8214 binds albumin and SSTR2, retaining octreotide-like potency

#### 3.2.1 TE-8214 is predominantly albumin-bound

**Figure 3:**
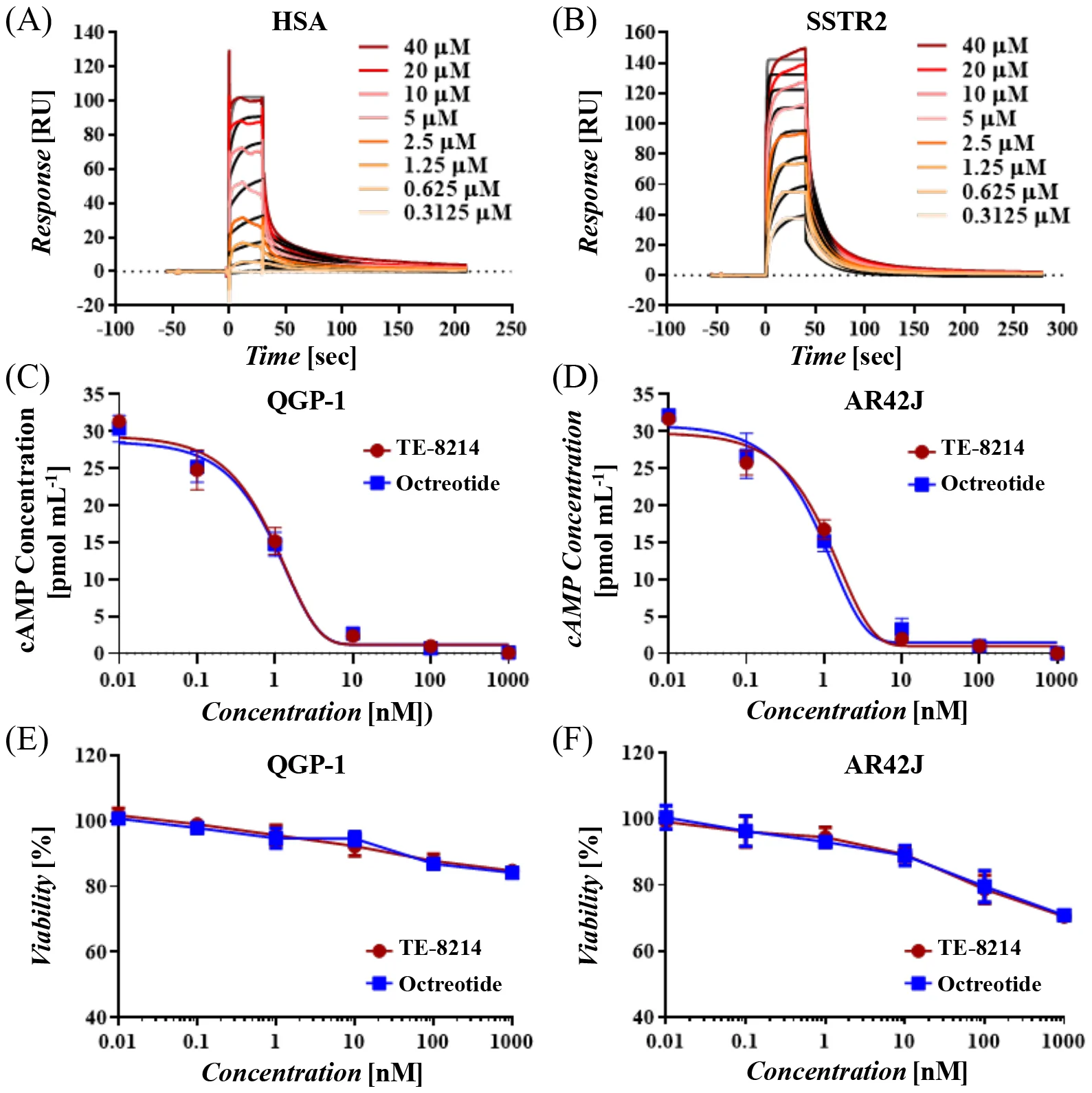
TE-8214 binds to HSA and SSTR2 and retains octreotide-like potency. SPR sensorgrams (response vs. time) showing concentration-dependent association/dissociation phases of TE-8214 with HSA **(A)** or SSTR2 **(B) **; fits to a 1:1 Langmuir model yielded dissociation constants (KD) of 1.86 mM forHSA-TE-8214 and 0.80 mM for SSTR2-TE-8214. Dose-dependent reduction of intracellular cAMP levels in QGP-1 **(C)** and AR42J **(D)** cells after a 20-minute incubation with TE-8214 or octreotide. Dose-dependent cell viability reductions in QGP-1 **(E)** and AR42J **(F)** cells after a 3-day incubation with TE-8214 or octreotide. Data are mean ± SD; n = 3.

To determine whether 2FA modification conferred albumin binding, we assessed TE-8214 binding to HSA by surface plasmon resonance (SPR). TE-8214 bound HSA with an association rate constant of 1.01 × 10^4^ M^–1^sec^–1^ and a dissociation rate constant of 1.88 × 10^−2^ sec^–1^, yielding a kinetic K_D_ of 1.86 μM (**Figure 3A**). Steady-state analysis of the same binding data gave an apparent K_D_ of 6.83 µM. The difference between the kinetic and steady-state affinity estimates is consistent with a binding interaction more complex than an ideal 1:1 model, potentially reflecting contributions from multiple FA-binding sites on HSA with differing affinities. The 1:1 Langmuir model applied to kinetic data provides an apparent affinity dominated by the major binding phase, whereas steady-state analysis reflects the integrated equilibrium response across the concentration range examined.

To determine the albumin-bound fraction of TE-8214, we evaluated the plasma protein-binding potential of TE-8214 in CD-1 mouse, SD rat, beagle dog, cynomolgus monkey, and human. Plasma protein binding measurements were performed using standardized equilibrium dialysis under physiological conditions (37 °C, pH 7.4) with triplicate determinations across five species. To determine the bound vs. unbound fractions of TE-8214, 0.5, 5, or 50 µM TE-8214 was dialyzed against PBS for 5 hours, allowing unbound drug to diffuse into the buffer chamber while protein-bound drug remained in the plasma chamber. LC-MS/MS quantification of post-dialysis TE-8214 concentrations in both chambers showed >99.99% protein-bound in all five species at all concentrations tested; free (unbound) concentrations were below the assay LLOQ (1 ng/mL). Semaglutide, a C18-conjugated GLP-1 receptor agonist, is similarly reported to be >99% bound to plasma albumin (PubChem CID 56843331) (22). Because HSA, the most abundant plasma protein, has a concentration (~0.6 mM (19)) vastly exceeding TE-8214 concentration, TE-8214 circulates predominantly in the albumin-bound state, creating a sustained drug reservoir. As the free drug is cleared from plasma, it is replenished via reversible dissociation of TE-8214 from albumin. This dynamic equilibrium functions as a systemic buffering mechanism, reducing renal filtration and enzymatic degradation, while sustaining receptor engagement and systemic exposure without local depot formation.

To confirm that the 2FA bundle enables albumin binding without compromising SSTR2 binding, we assessed TE-8214 binding to SSTR2 by SPR. TE-8214 bound SSTR2 with a K_D_ of 0.80 μM (**Figure 3B**). Next, we investigated whether TE-8214 binding SSTR2 translated into functional potency comparable to the parent drug, octreotide, by monitoring SSTR2 activation through both mechanistic and clinically relevant biomarkers in SSTR2-expressing cells. Intracellular cAMP is a mechanistic readout of SSTR2 activation (23–25). SSAs inhibit adenylate cyclase, reducing cAMP levels, leading to suppression of hormone secretion (1, 26, 27). Serotonin contributes to carcinoid symptoms and is used clinically to monitor functioning NETs (27, 28); CgA is a widely used pan-NET marker of tumor burden and neuroendocrine differentiation (29–31). Assays were conducted in QGP-1 human pancreatic NET cells and AR42J rat pancreatic tumor cells, both established SSTR2-postive models for studying SSA activity (32–35).

In SSTR2-expressing cell-based assays conducted in the presence of bovine-derived albumin, TE-8214 matched octreotide’s activity across mechanistic and disease-relevant readouts: TE-8214 reduced cAMP with EC_50_ values of 0.74 ± 0.40 nM (QGP-1 cells, **Figure 3C**) and 1.00 ± 0.13 nM (AR42J cells, **Figure 3D**) comparable to octreotide (0.85 ± 0.12 and 0.72 ± 0.18 nM). It suppressed serotonin secretion in QGP-1 cells and CgA expression in AR42J cells with potency similar to octreotide (**Supplementary Figures 7A, B**). Furthermore, TE-8214 reduced cell viability in a dose-dependent manner across four SSTR-expressing cell lines, closely mirroring octreotide’s activity (**Figures 3E, F**; **Supplementary Figures 7C, D**).

Thus, the *in vitro* functional assays demonstrate no significant efficacy difference between TE-8214 and octreotide, suggesting that the 2FA module confers albumin binding without impairing SSTR2 binding affinity or SSTR2-mediated function.

### 3.3 TE-8214’s albumin binding extends exposure and efficacy *in vivoc*

#### 3.3.1 Albumin binding extends TE-8214 exposure across species

**Figure 4:**
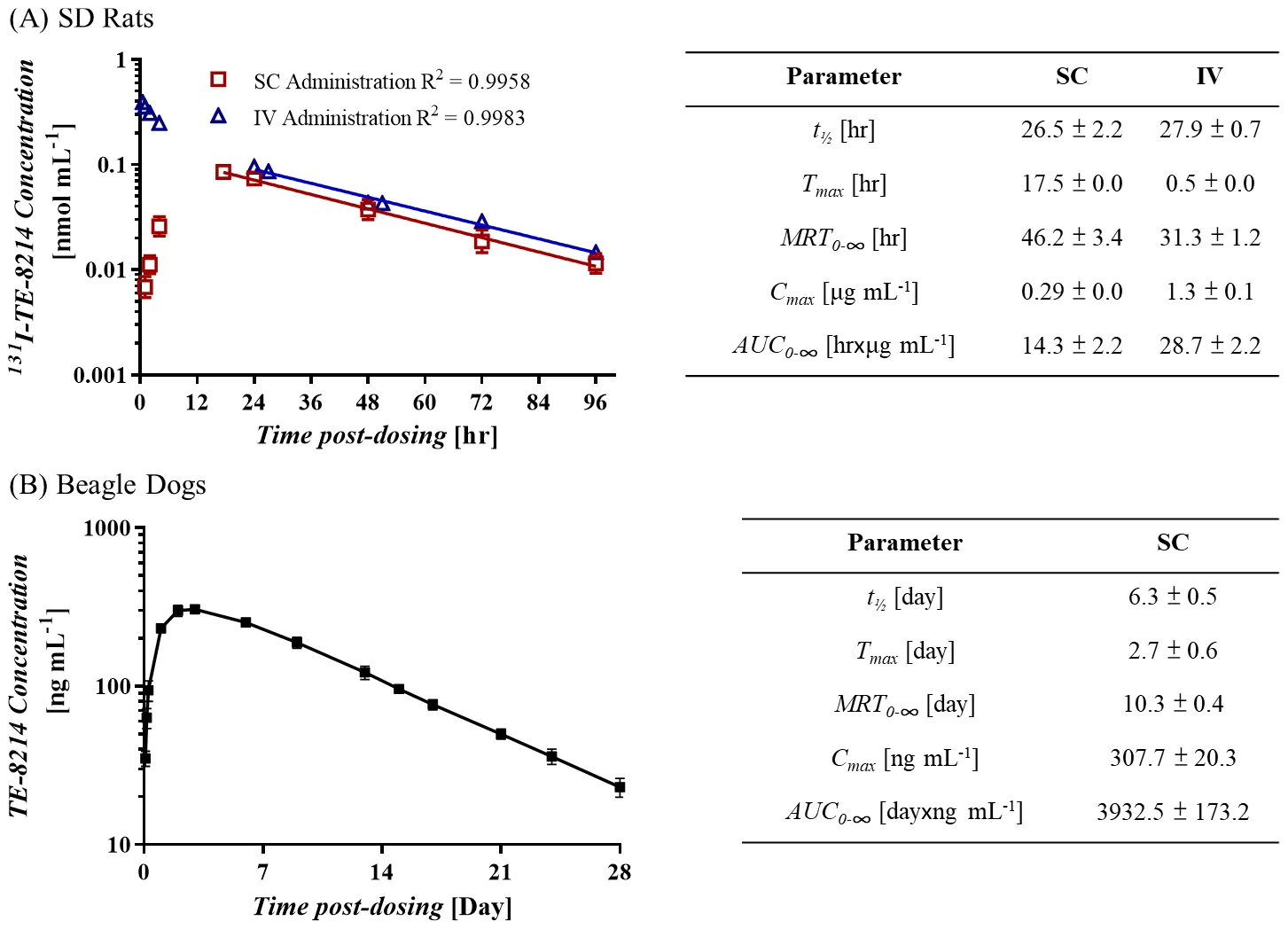
Prolonged terminal half-life of TE-8214 in rats and dogs. **(A)** SD rats (n = 3) received a single SC (124.5 μg kg^-1^) or IV (102.6 μg kg^-1^) injection of ^131^I-TE-8214; plasma levels were measured by gamma counting. **(B)** Beagle dogs (n = 3) received a single SC TE-8214 dose (40 µg kg^-1^); plasma levels were quantified by validated LC-MS/MS. Pharmacokinetics parameters were calculated by non-compartmental analysis using PKSolver. *T_max_* is the time to reach the maximum plasma concentration *C_max_*; *MRT*_0–__∞_ is the mean residence time; and *AUC*_0–__∞_ is the total area under curve from time 0 to infinity. Data are mean ± SD; n = 3.

To assess whether albumin binding extends *in vivo* circulation time, we evaluated the pharmacokinetics of TE-8214 in SD rats and beagle dogs. Independent of the administration route in rats, TE-8214’s terminal half-life (t_1/2_) was 27−28 hours (**Figure 4A**), much longer than FA-free octreotide’s *t_1/2_* of ~20 minutes in rats (36). In dogs, a single SC dose of TE-8214 yielded a *t_1/2_* of 6.3 ± 0.5 days (**Figure 4B**), but the t_1/2_ for FA-free octreotide in dogs could not be found in the current literature. However, the rapid clearance of octreotide across species, ~20 mins in rats to ~100 mins in humans (8, 9), suggests that conjugating 2FAs to octreotide in TE-8214 consistently extended half-life.

#### 3.3.2 TE-8214 delivers durable hormone suppression and outlasts equimolar octreotide

**Figure 5:**
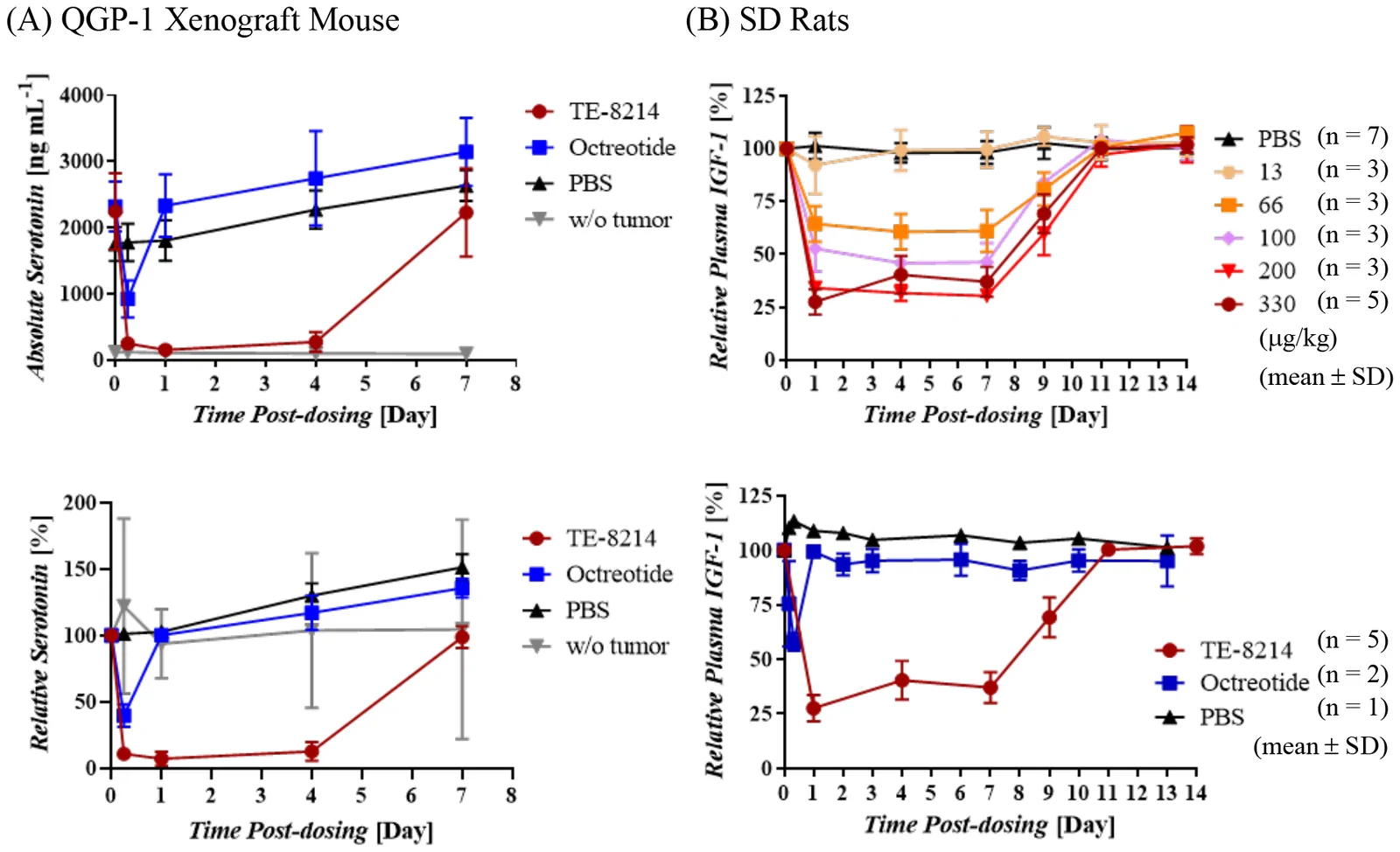
TE-8214 outlasts equimolar octreotide in hormone suppression. **(A)** Plasma serotonin (absolute, top panel and % pre-dose baseline, bottom panel) in NOD SCID mice with/without QGP-1 tumors after a single SC dose of TE-8214 (0.34 mg kg^-1^), equimolar octreotide (0.11 mg kg^-1^), or PBS. Data are mean ± SD; n = 3–4 per group. **(B)** Top panel: Plasma IGF-1 (% pre-dose baseline) in rats after a single SC dose of TE-8214 (13–330 μg kg^-1^). The lowest dose (13 μg kg^-1^) was ineffective in suppressing plasma IGF-1, while the highest dose (330 μg kg^-1^) provided no statistically significant additional suppression of plasma IGF-1 compared to the 200 μg kg^-1^ dose (p = 0.14). Bottom panel: Plasma IGF-1 (% pre-dose baseline) in rats after a single SC dose of TE-8214 (330 μg kg^-1^, red) or equimolar octreotide (100 μg kg^-1^, blue) or PBS (black). TE-8214 (330 μg kg^-1^) provides durable IGF-1 suppression, whereas equimolar octreotide (100 μg kg^-1^) is effective for <24 hours. Data are mean ± SD; group sizes are indicated in the panels.

We next assessed whether prolonged exposure translates into sustained pharmacodynamics by comparing the ability of single, equimolar doses of TE-8214 and octreotide to suppress serotonin and IGF-1, key biomarkers of functioning NETs and acromegaly, respectively, in animal models. At equimolar doses, TE-8214 outlasted octreotide in suppressing these disease-relevant biomarkers: In QGP-1 xenograft–bearing mice, a single SC dose of TE-8214 (0.34 mg kg^-1^) suppressed plasma serotonin to ~10% of baseline through Day 4 (**Figure 5A**). In SD rats, TE-8214 suppressed IGF-1 to 28−64% of baseline for 7 days across effective doses (66–330 μg kg^-1^), with recovery by Day 11 (**Figure 5B**). In contrast, equimolar octreotide was ineffective beyond 24 hours for both biomarkers.

#### 3.3.3 TE-8214 exhibits clear exposure-response relationship in dogs

**Figure 6:**
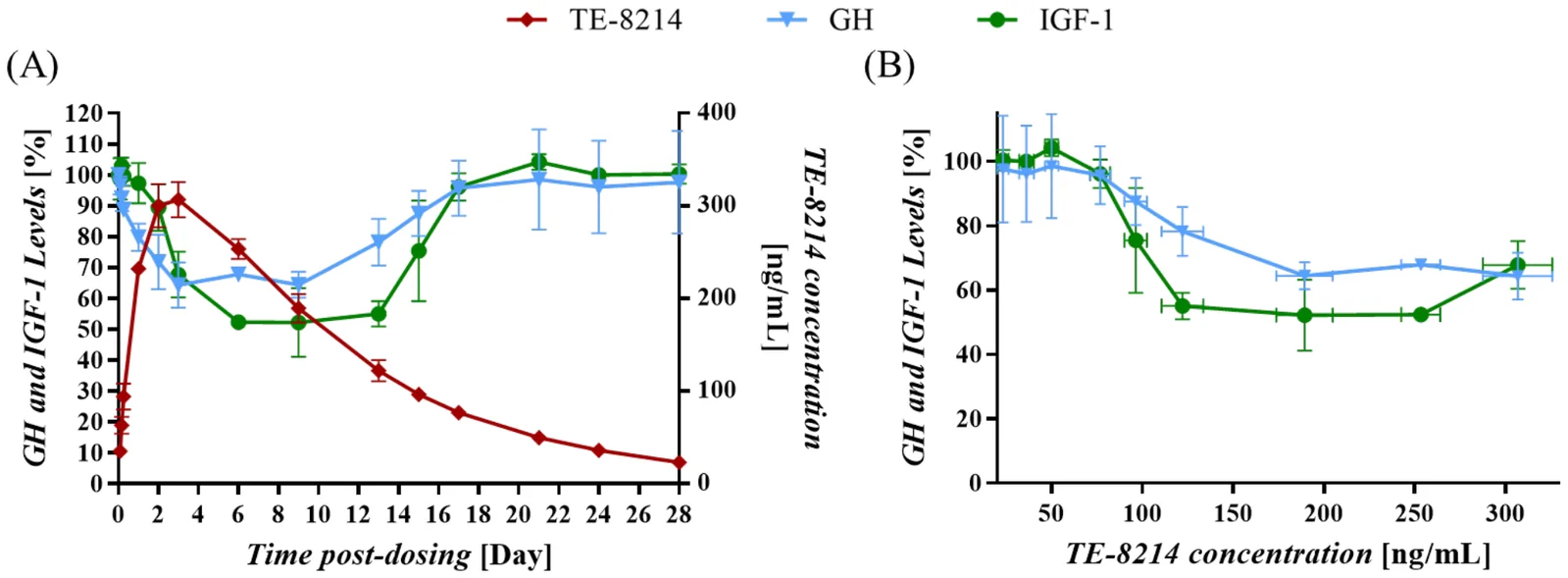
Exposure-response in dogs after a single TE-8214 dose. **(A)** Plasma TE-8214 concentration and corresponding growth hormone and IGF-1 levels following a single subcutaneous dose of TE-8214 (40 µg kg^-1^). **(B)** Concentration–response plots of total plasma TE-8214 concentration versus GH and IGF-1, expressed as percentages of their respective predose baselines. Data are mean ± SD; n = 3.

To confirm pharmacokinetics/pharmacodynamics linkage, we measured GH and IGF-1 levels after a single SC dose of TE-8214 (40 μg kg^-1^) in beagle dogs. TE-8214 reduced GH to 64–78% and IGF-1 to 52–75% of baseline for 11 and 13 days, respectively, returning to >95% of baseline by Day 17, in parallel with declining drug concentrations (**Figure 6A**). Plotting GH and IGF-1 levels against corresponding TE-8214 concentration (albumin-bound plus free) revealed a clear exposure-response relationship (**Figure 6B**). No adverse effects or abnormalities in cage-side observations, body weight, body temperature, or clinical pathology were found. These results demonstrate a temporal association between sustained TE-8214 exposure and multi-day endocrine suppression.

#### 3.3.4 Chronic TE-8214 dosing maintains stable biomarker suppression

**Figure 7:**
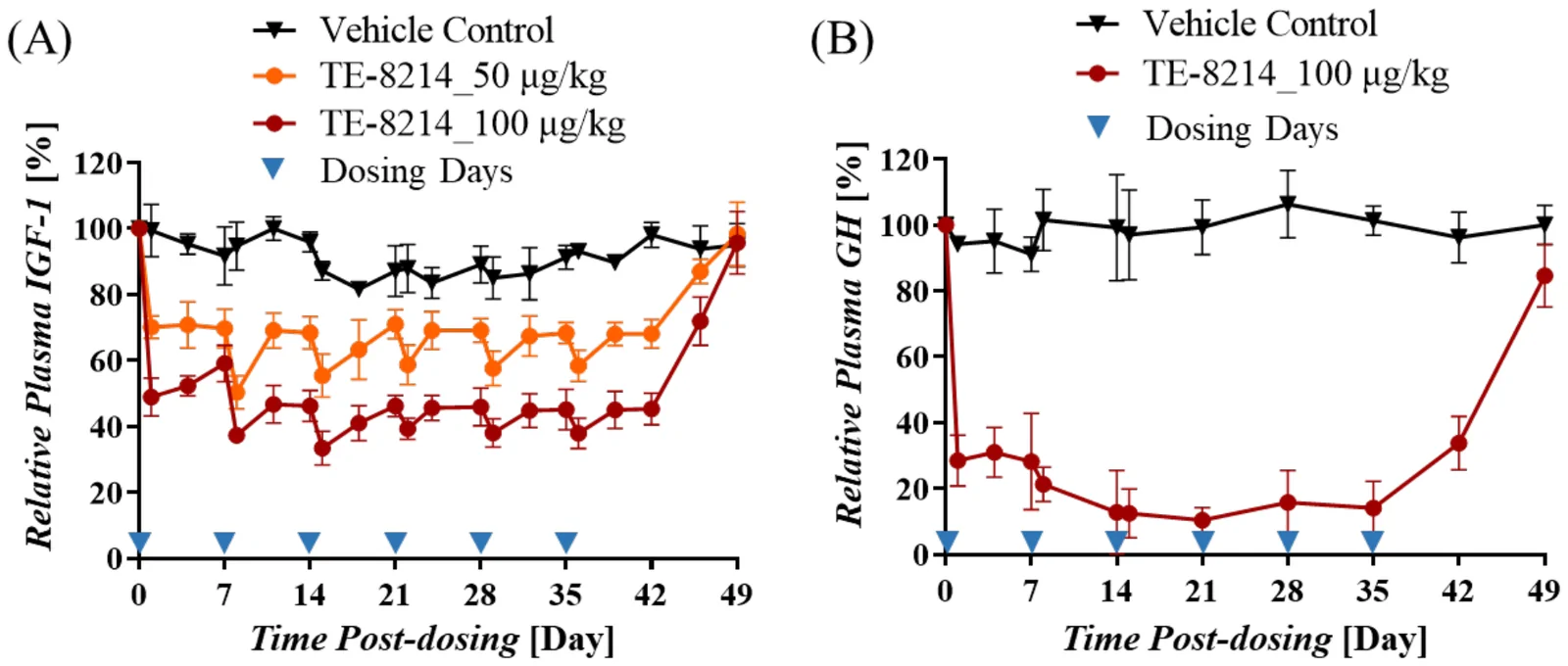
Sustained IGF-1 and GH suppression with once-weekly TE-8214 in rats. **(A)** Relative plasma levels of IGF-1 in SD rats receiving six once-weekly SC injections of vehicle or TE-8214 (50 or 100 μg kg^−1^). Data are mean ± SD; n = 4. **(B)** Relative plasma levels of GH in SD rats receiving six once-weekly SC injections of vehicle or TE-8214 (100 μg kg^−1^). Data are mean ± SD; n = 3.

Because long-acting therapies must sustain effects under chronic dosing, we tested once-weekly SC administration of TE-8214 in SD rats. Over six weeks, once-weekly SC injections of TE-8214 (50 or 100 μg kg^-1^) maintained stable IGF-1 and GH suppression throughout the entire 42-day treatment window. At 100 μg kg^-1^, IGF-1 was suppressed to 34–59% of baseline (**Figure 7A**) and GH levels to 10–34% of baseline for 42 days (**Figure 7B**), before returning to baseline on Day 49. Body weight, blood glucose, and clinical chemistry remained within normal ranges.

### 3.4 TE-8214 is safe and well-tolerated in SD rats and beagle dogs

To determine the starting dose for clinical human studies, Good Laboratory Practice (GLP)-compliant 4-week repeated dose toxicity studies were conducted in SD rats with twice weekly dosing and beagle dogs with once weekly dosing. All animals survived the dosing and recovery periods. The NOAEL was determined to be 18 mg kg^−1^ in SD rats and 2.5 mg kg^−1^ in beagle dogs (see below).

#### 3.4.1 SD rats

TE-8214-related effects were primarily pharmacological, including non-adverse decreases in body weight, food consumption, and serum IGF-1 levels, all of which reversed during recovery. Microscopic injection-site findings were limited to minimal-to-mild mixed-cell inflammation and hemorrhage that occurred in both vehicle- and TE-8214-treated animals and largely resolved during recovery (**Supplementary Table 1**), consistent with the administration procedure rather than a drug-related effect. In-life local irritation scores for edema and erythema were 0 in all animals throughout dosing and recovery (**Supplementary Table 2**). No systemic toxicity was identified. Toxicokinetic analysis showed dose-dependent exposure without gender differences, with an accumulation ratio of 1.4–1.7 by Day 26 (**Supplementary Table 3**). Under these conditions, the NOAEL for TE-8214 in SD rats was established at 18 mg kg^−1^, the highest dose tested, corresponding to a mean steady-state *AUC_0-t_* of 5.1 × 10^6^ h×ng mL^−1^ in males and 5.8 × 10^6^ h×ng mL^−1^ in females.

#### 3.4.2 Dogs

No TE-8214-related effects on body weight, food consumption, or clinical pathology were found. Treatment-related signs were limited to transient soft feces and non-adverse ECG changes primarily at doses ≥2.5 mg kg^−1^, which reversed after recovery. Toxicokinetic analysis showed dose-dependent exposure without gender differences, with an accumulation ratio of 1.8–1.9 by Day 22 (**Supplementary Table 4**). While dose-dependent lymphoid hypocellularity in the thymus and minimal hepatic changes were considered non-adverse, splenic congestion at the 7.0 mg kg^−1^ dose was deemed potentially adverse (**Supplementary Table 5**). In-life local irritation scores for edema and erythema were 0 in all dogs throughout dosing and recovery (**Supplementary Table 6**). Consequently, the NOAEL was established at 2.5 mg kg^−1^, corresponding to a mean steady-state *AUC_(0-168h)_* of ~3.8 × 10^6^ h×ng mL^−1^.

Safety margins were calculated by converting the animal NOAELs to human equivalent doses with FDA allometric factors and comparing these with the maximum human dose of 4 mg (0.067 mg kg^−1^ based on a 60 kg body weight). The resulting margins were 43.3-fold for the rat and 20.9-fold for the dog, demonstrating a favorable safety profile (**Supplementary Table 7**).

TE-8214 was also evaluated for genotoxicity and safety pharmacology. TE-8214’s genotoxicity was assessed using Ames test (using *Salmonella Typhimurium*) to detect gene mutations and *in vitro* mammalian chromosomal aberration assay (using Chinese hamster lung fibroblast cells) as well as *in vivo* bone marrow micronucleus test (in SD rats) to detect chromosomal damage. TE-8214 was not considered genotoxic, showing negative findings in all 3 tests. TE-8214’s safety pharmacology profile was established via (i) *in vitro* human ether-à-go-go-related gene assay, (ii) *in vivo* cardiovascular assessment in beagle dogs, and (iii) standalone central nervous system and respiratory studies in SD rats. TE-8214 demonstrated no significant adverse effects in all these safety pharmacology studies.

### 3.5 TE-8214 was well-tolerated and sustained IGF-1 reduction in healthy adults

We conducted a randomized, double-blind, placebo-controlled single-ascending-dose Phase 1 study of TE-8214 (TE-8214-001) in 32 healthy adults to evaluate the safety, tolerability, and pharmacokinetics. Pharmacodynamics effect was also assessed using IGF-1, a validated, regulatory-accepted translational biomarker for SSA activity. First-in-Human studies in healthy volunteers are in accordance with European Medicines Agency’s guideline on strategies to mitigate risks with investigational drugs, prior to evaluation of repeated dosing in patient populations. A single-ascending-dose design was implemented instead of a multiple-ascending dose design, as TE-8214-induced long-term reductions in GH and IGF-1 levels may cause unnecessary risks for healthy volunteers. All 32 participants completed the trial: 24 received TE-8214 at escalating single SC doses (0.6, 1.2, 2.0, or 4.0 mg; 6 per cohort) and 8 received saline placebo, all via fine 30-gauge needle injection in the abdomen. Dose selection was guided by preclinical toxicology studies in rats and dogs, providing a ≥20-fold safety margin at the highest dose of 4 mg, consistent with standard practices for first-in-human studies.

#### 3.5.1 TE-8214 was well-tolerated across all dose levels

No deaths, serious adverse events (SAEs), or Grade ≥3 TEAEs occurred in any cohort (**Supplementary Table 8**). Tolerability of TE-8214 was similar to that of placebo, supporting the feasibility of fine-needle administration: Overall TEAE incidence was comparable between participants receiving TE-8214 (70.8%) and placebo (87.5%). No injection-site pain, pruritus (itching), induration, and nodule formation, which are the major reactions for long-acting SSAs, were reported; only transient injection-site bruising and erythema occurred, lasting 1−2 days (**Supplementary Table 9**). These findings are consistent with the absence of adverse effects/abnormalities found in dogs or during repeated dosing in rats.

#### 3.5.2 TE-8214 prolonged circulation

Single-dose administration of TE-8214 resulted in dose-dependent increases in systemic exposure across the evaluated dose range, as evidenced by the *C_max_* and *AUC_0-∞_* values (**Table 1**). *C_max_* variability, quantified by coefficients of variation (*CV*%) computed as SD/mean × 100, decreased from 42.1% at 1.2 mg to 18.5% at 4 mg. TE-8214’s mean terminal half-life (*t_1/2_* = 91−131 hours) was prolonged relative to native octreotide’s half-life (1.67 hours), consistent with the albumin-mediated reduction in renal filtration and proteolytic degradation described in Section 4.2, as reflected in the low apparent clearance (*CL/F*). At the highest human dose (4.0 mg), TE-8214’s half-life (131.0 ± 32.6 hours, i.e., 5.5 ± 1.4 days) overlapped with the canine estimate (6.3 ± 0.5 days) within experimental error (**Figure 4B**). The difference at lower human doses may reflect differences in dose, sample size, sampling schedule, and estimation of the terminal elimination slope between the canine and human studies. However, subtle species-dependent differences in albumin interaction or clearance cannot be excluded because unbound TE-8214 concentrations were below the LLOQ.

**Table 1:** Pharmacokinetic parameters of plasma TE-8214 from the Phase 1 study.

Parameter^a^	0.6 mg (N = 6)	1.2 mg (N = 6)	2.0 mg (N = 6)	4.0 mg (N = 6)
*C_max_* [ng mL^−1^]	44.6 ± 13.4	105 ± 44.2	168 ± 50.7	325 ± 60.1
CV [%]	30.0	42.1	30.2	18.5
*T_max_* [hr]	84 (48−144)	73 (48−144)	85 (48−149)	97 (47−147)
*AUC*_0–*t*_ [hr×ng mL^−1^]	9550 ± 1730	24200 ± 8550	42200 ± 10800	108000 ± 26600
*AUC*_0–∞_ [hr×ng mL^−1^]	9760 ± 1740	24600 ± 8660	42700 ± 11000	114000 ± 31600
*AUC_extrap_* [%]	2.22 ± 0.67	1.85 ± 0.675	1.23 ± 0.442	4.99 ± 2.75
*t_1/2_* [hr]	93.7 ± 9.9	91.6 ± 11.7	90.7 ± 8.7	131.0 ± 32.6
*λ_z_* [hr^−1^]	0.0075 ± 0.0008	0.0077 ± 0.0010	0.0077 ± 0.0008	0.0056 ± 0.0014
*CL/F* [L hr^−1^]	0.063 ± 0.011	0.054 ± 0.019	0.049 ± 0.012	0.037 ± 0.009
*V_z_/F* [L]	8.55 ± 1.77	7.09 ± 2.47	6.42 ± 1.57	6.67 ± 0.574
*C_max_/D* [ng mL^−1^ mg^−1^]	74.4 ± 22.3	87.2 ± 36.8	83.8 ± 25.4	81.2 ± 15
*AUC*_0–*t/D*_ [hr×ng mL^−1^ mg^−1^]	15900 ± 2880	20200 ± 7130	21100 ± 5410	27000 ± 6640
*AUC*_0–∞_/*D* [hr×ng mL^−1^ mg^−1^]	16300 ± 2890	20500 ± 7220	21400 ± 5490	28600 ± 7900

^a^N = Total number of participants in each group; *T_max_* is the time to reach the maximum plasma concentration *C_max_*; *AUC*_0–∞_ is the total area under the plasma concentration–time curve from time 0 to infinity; t_1/2_ is the terminal half-life; *CL/F* is the apparent clearance. Data are mean ± standard deviation (N = 6), except for *T_max_*, presented as median (range).

#### 3.5.3 TE-8214 produced sustained, dose-dependent reductions in plasma IGF-1

**Figure 8:**
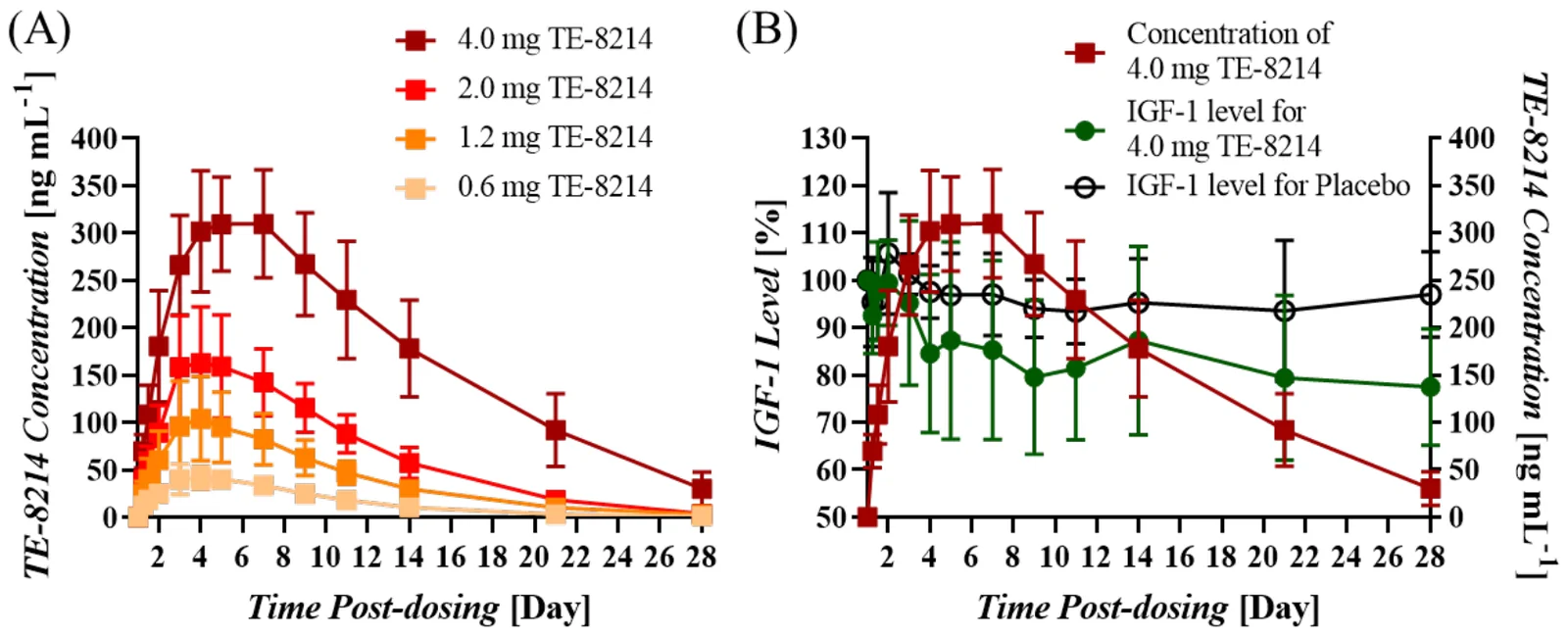
Prolonged pharmacokinetics and dose-dependent IGF-1 reductions in humans. **(A)** Plasma TE-8214 concentrations were quantifiable from 6 hours through Day 28, with exposure increasing across ascending single SC doses (0.6−4.0 mg). **(B)** Sustained exposure translated into durable, dose-dependent IGF-1 reductions.

The increase in TE-8214’s plasma concentration with ascending doses (**Figure 8A**) correlated with progressively greater IGF-1 suppression. Longitudinal IGF-1 percentage changes from baseline were analyzed using a mixed model two-way ANOVA. At Day 28, TE-8214 at 4.0 mg reduced IGF-1 by 22.5% levels relative to baseline (mean change −22.5%; p = 0.0367, **Figure 8B**), whereas the placebo group yielded no significant IGF-1 changes (+5.7% to −7.7%). Importantly, suppression was durable: IGF-1 remained ≥12% below baseline from Day 4 through Day 28 for the 4.0 mg group. This prolonged effect mirrors the prolonged IGF-1 suppression observed in rats and dogs.

GH was also assessed in each participant; however, concentrations were 0.1 μg L^−1^ regardless of TE-8214 treatment, precluding any meaningful assessment of a treatment effect. Because basal GH in healthy adults is characteristically low (typically <5 μg L^−1^ in men and < 10 μg L^−1^ in women) and secreted in discrete pulses, often peaking during sleep, evaluation of TE-8214’s effect on GH will be more informative in patients with acromegaly, whose chronically elevated GH concentrations provide a measurable baseline for suppression.

## 4 Discussion

In 1991, one of the authors (T. W. Chang) in this study had proposed extending the pharmacokinetics of therapeutic proteins and peptides via site-specific conjugation of lipophilic moieties such as FAs (37). However, its application to octreotide to achieve sustained systemic exposure and pharmacodynamic activity in humans from a fine 30-gauge needle injection had not been accomplished. For a small octapeptide such as octreotide, which lacks the additional backbone stabilizing substitutions used in lipidated GLP-1 analogs such as semaglutide and tirzepatide, a single FA attached to a Lys may provide insufficient albumin affinity for clinically meaningful half-life extension while maintaining aqueous solubility. However, conjugating 2FAs to octreotide to enhance albumin affinity is non-trivial: it is challenging to match the FA characteristics (different lengths, terminal groups, and spacing) to albumin’s seven FA-binding pockets, which vary in shape, depth, and electrostatic properties. TE-8214 utilizes a soluble C16-CH_3_/C18-COO^−^ 2FA bundle that provided better hormone suppression than homogeneous C16/C18/C20 diacids (**Supplementary Figure 3**). Furthermore, the (PEG_4_Lys)_2_ linker facilitates an aqueous, low-viscosity formulation, enabling TE-8214 to remain fully soluble post-injection.

TE-8214 is the first 2FA-conjugated octreotide analog to demonstrate sustained, multi-day human exposure and 28-day IGF-1 suppression from a single aqueous, fine-needle SC injection, without relying on depot formulations. All approved long-acting SSAs such as octreotide LAR and Lanreotide Autogel rely on depot formation to achieve sustained exposure. TE-8214 may therefore mitigate the trade-off between exposure durability and patient comfort: its aqueous formulation enabled fine 30-gauge needle SC administration with tolerability comparable to saline placebo in the Phase 1 study. Below, we discuss (i) the mechanistic basis of prolonged exposure via reversible albumin binding, (ii) the clinical implications of fine-needle aqueous delivery relative to depot formulations, (iii) the versatility of the modular 2FA linker and (iv) the limitations of the first-in-human dataset.

TE-8214’s pharmacokinetic profile is consistent with a clearance-limited mechanism mediated by reversible albumin binding, rather than absorption-limited depot release. *In vivo*, endogenous FAs dynamically occupy albumin’s FA-binding sites and could, in principle, compete with TE-8214. However, because albumin circulates at ~0.6 mM, several orders of magnitude above circulating TE-8214 concentrations, even substantial endogenous FA occupancy is unlikely to meaningfully reduce the fraction of albumin available for TE-8214 binding. Thus, TE-8214 remains predominantly albumin-bound (see section 4.2), which reduces renal filtration (low *CL/F*, **Table 1**) and enzymatic degradation, thereby extending systemic terminal half-life. Sustained pharmacodynamic activity (**Figure 8B**) is consistent with continuous replenishment of free drug via reversible dissociation from the circulating albumin-bound reservoir, supporting prolonged receptor engagement over the dosing interval. The observed rapid drug absorption, absence of a secondary absorption phase (**Figure 8A**) and absence of injection-site nodules are consistent with this clearance-limited, albumin-buffered mechanism.

TE-8214 differs from approved long-acting SSAs (2) primarily in formulation and proposed mechanism of prolonged exposure. Unlike fixed-dose depot systems, TE-8214’s low-viscosity aqueous formulation may permit dose adjustment by injection volume using a fine 30-gauge needle. Compared to published data for octreotide depots after one injection in healthy volunteers (38), TE-8214 showed a lower inter-individual variability in *C_max_* (lower %*CV*), but comparable terminal half-life and extent of IGF-1 reduction to within experimental error (**Table 2**). Oral octreotide (Mycapssa™) requires twice-daily dosing in a fasted state (39), which poses long-term adherence challenges and risks loss of hormonal control if a dose is missed (2, 40). In contrast, TE-8214 is administered subcutaneously and is therefore not subject to fasting requirements.

**Table 2:** TE-8214 compared to octreotide LAR and octreotide subcutaneous (sc) depot after 1 injection in healthy subjects.

Parameter	TE-8214^a^	Octreotide LAR^b^	Octreotide sc depot^b^
Dose	4.0 mg	30 mg	30 mg
Formulation	aqueous solution	depot microspheres	Liquid-crystal gel
Needle	30G	19G	22G
*C_max_* [ng mL^−1^]^c^	325 ± 60.1	1.05 ± 0.92	29.13 ± 9.75
*CV* [%]	18.5	87.6	33.5
*T_max_* [hr]	97 (47–147)	1.0 (0.5 – 359.9)	24.0 (2.0 – 49.2)
*t_1/2_* [hr]	131.0 ± 32.6	NA^d^	151 ± 60.6
% IGF-1 reduction from baseline	22.5 ± 12.3	35.7 ± 11.2	43.3 ± 12.3

aTaken from **Table 1** and **Figure 8**. ^b^Values are mean ± standard deviation (n = 13), except *T_max_*, presented as median (range); all data are taken from Tiberg et al, 2015 (38). ^c^*C_max_* values represent total (protein-bound plus free) drug concentrations. Because TE-8214 is >99.99% plasma-protein bound across five species (Section 4.2), whereas octreotide is only ~65% plasma-protein bound, these total concentration values are not directly comparable in terms of receptor-available (free) drug. ^d^NA denotes not applicable.

A key strength of the 2FA strategy lies in its consistent pharmacokinetic and pharmacodynamic behavior across species, supporting translational continuity from preclinical models to humans. The 2FA strategy consistently extended drug half-life, produced durable pharmacodynamic effects, and demonstrated favorable safety across rodents, dogs, and humans, supporting continued clinical development. The same 2FA strategy with (C16-CH_3_/C18-COO^−^) attached to a (PEG_4_Lys)_2_ linker had been applied to develop TE-8105, a long-acting GLP-1 receptor agonist. TE-8105 demonstrated enhanced preclinical efficacy in glycemic control, weight loss, and liver health compared with semaglutide (15), and met safety, tolerability and pharmacokinetics endpoints in a Phase 1/2a trial in overweight/obese participants (NCT06471530). No PEG-related safety signals were identified in the clinical studies of TE-8214 or TE-8105. The parallel success of TE-8214 (oncology) and TE-8105 (metabolic disease) supports the utility of the modular 2FA linker for designing long-acting peptide therapeutics.

This study has several limitations. First, the non-clinical pharmacology studies were exploratory, unrandomized, and unblinded, with small group sizes, and are therefore hypothesis-generating rather than confirmatory. Second, because unbound TE-8214 concentrations were below the assay LLOQ, exposure–response relationships could not be tied to free-drug concentrations. Third, the clinical investigation involved healthy volunteers rather than patients with acromegaly or neuroendocrine tumors, thus direct efficacy comparisons with approved depot SSAs cannot be made. Fourth, in the first-in-human study, only single-dose administration was evaluated, precluding assessment of steady-state pharmacokinetics, long-term safety, or sustained therapeutic efficacy, while the modest cohort size precluded reliable sex/gender-, or age-stratified analyses of safety, pharmacokinetics, or pharmacodynamics. Fifth, although IGF-1 provided evidence of pharmacodynamic target engagement, it does not capture the serotonin- or chromogranin A-mediated symptoms characteristic of functioning NETs. The last three limitations will be assessed in a planned multiple-ascending dose, repeated dosing, Phase 2 trial in patients with NETs to evaluate long-term safety and efficacy including tumor imaging and effects on NET-specific biomarkers.

In summary, TE-8214 exhibited prolonged systemic exposure and sustained exploratory IGF-1 reduction after a single subcutaneous dose in healthy adults. No SAEs or Grade ≥3 TEAEs were observed in this Phase 1 study. Together with the preclinical findings, these results support further clinical evaluation of TE-8214 in patients with neuroendocrine tumors or acromegaly.

## Data Availability

The original contributions presented in the study are included in the article/**Supplementary Material**. Further inquiries can be directed to the corresponding authors.
