# Supplementary material for: A long-acting octreotide analog formed by conjugation with a peptide linker modified with two fatty acids
Source: Front Endocrinol (Lausanne). 2026 Aug 25;17:1871977. doi: 10.3389/fendo.2026.1871977 (PMC13551246; doi:10.3389/fendo.2026.1871977)
Supplement: Supplementary file 1 [file SupplementaryFile1.pdf]

Supporting Information

for

A Long-Acting Octreotide Analog Formed by Conjugation with a Peptide Linker Modified with Two Fatty Acids

Yueh-Hsiang Yu,[a]* Ya-Shan Chuang,[b] Jessica Gehlert,[c] Chi-Jiun Peng,[a] Wei-Chen Lin,[a] Mun-Teng Wong,[b] Jon Wright,[b] Hsing-Mao Chu,[a,b] Carmay Lim,[a,b]* and Tse Wen Chang,[a,b]*

[a]T-E Meds, Inc., C423, No. 99, Lane 130, Academia Road, Section 1, Nangang, Taipei 115021

[b]Immunwork, Inc., C520, No. 99, Lane 130, Academia Road, Section 1, Nangang, Taipei 115021, Taiwan

[c]CMAX Clinical Research - Level 5, 18a North Terrace; Adelaide SA 5000, Australia

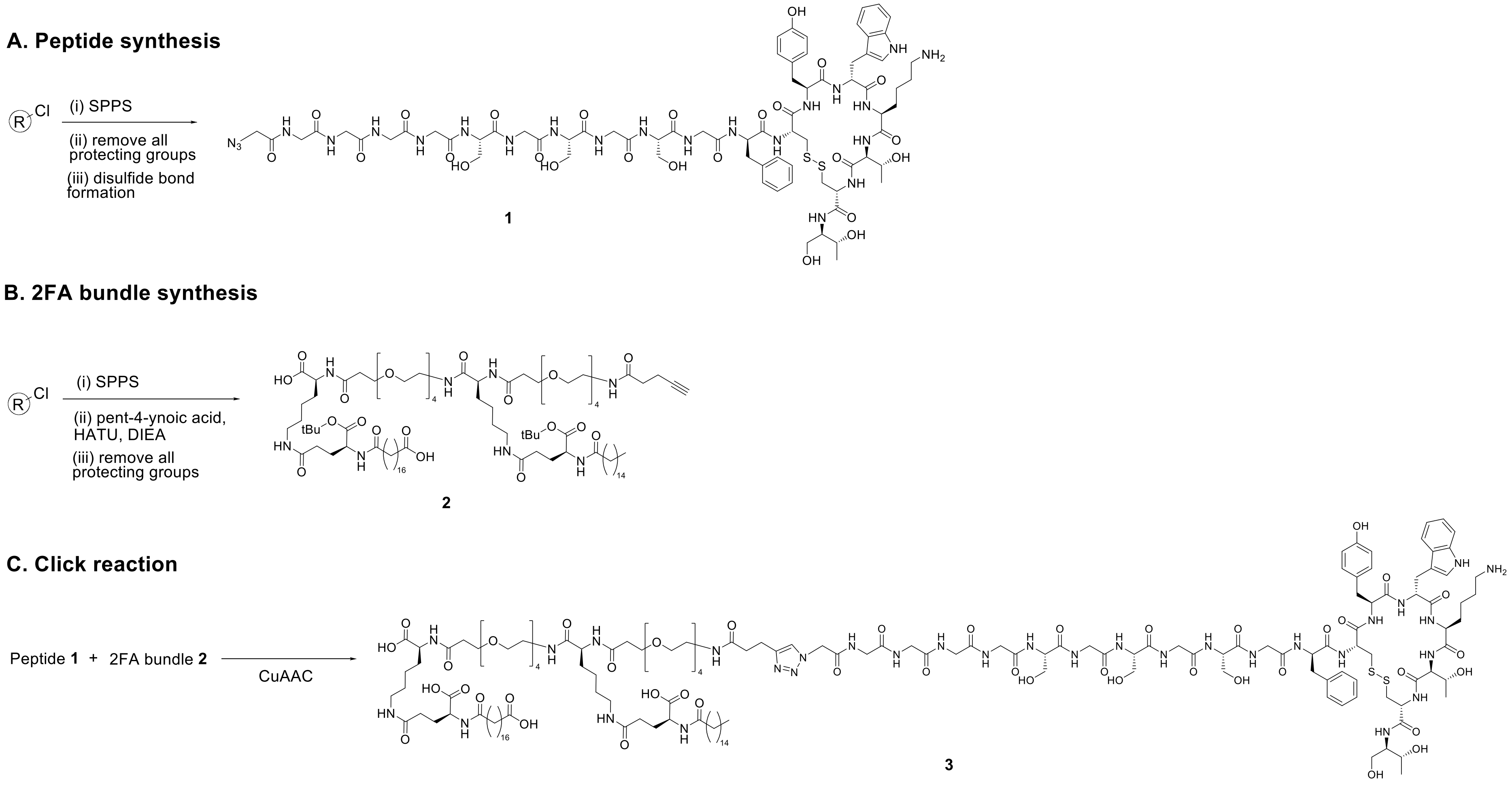

Supplementary Figure S1. Synthesis of TE-8214 Using a Multi-arm Linker

B. 2FA bundle synthesis

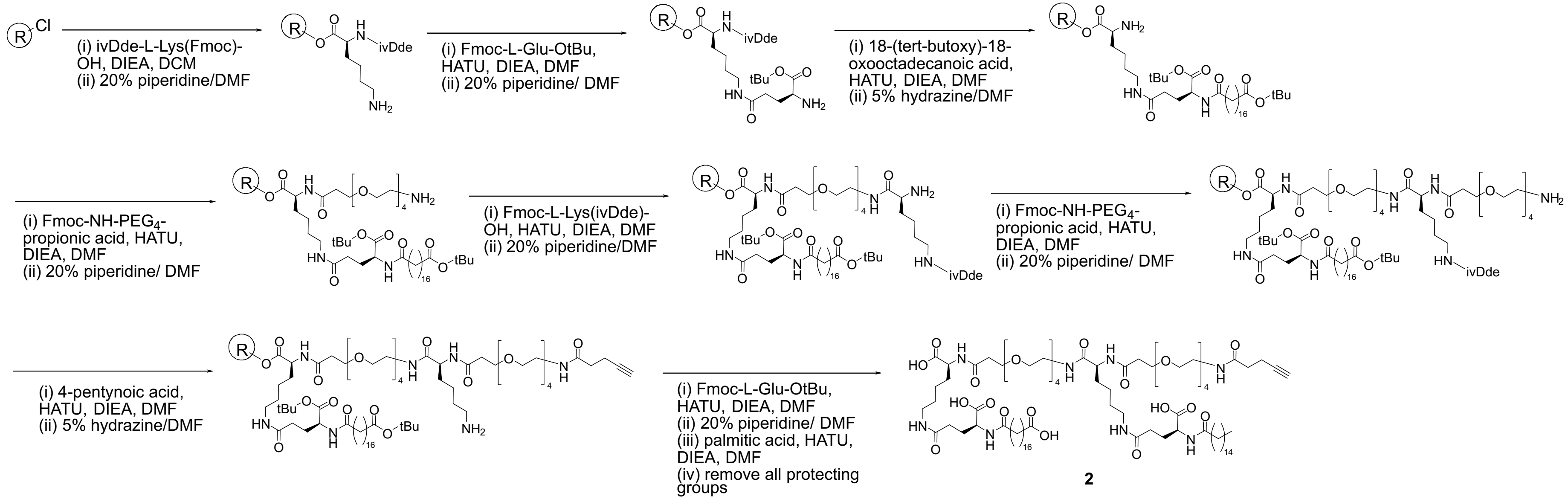

Supplementary Figure S2. Synthesis of 2FA bundle

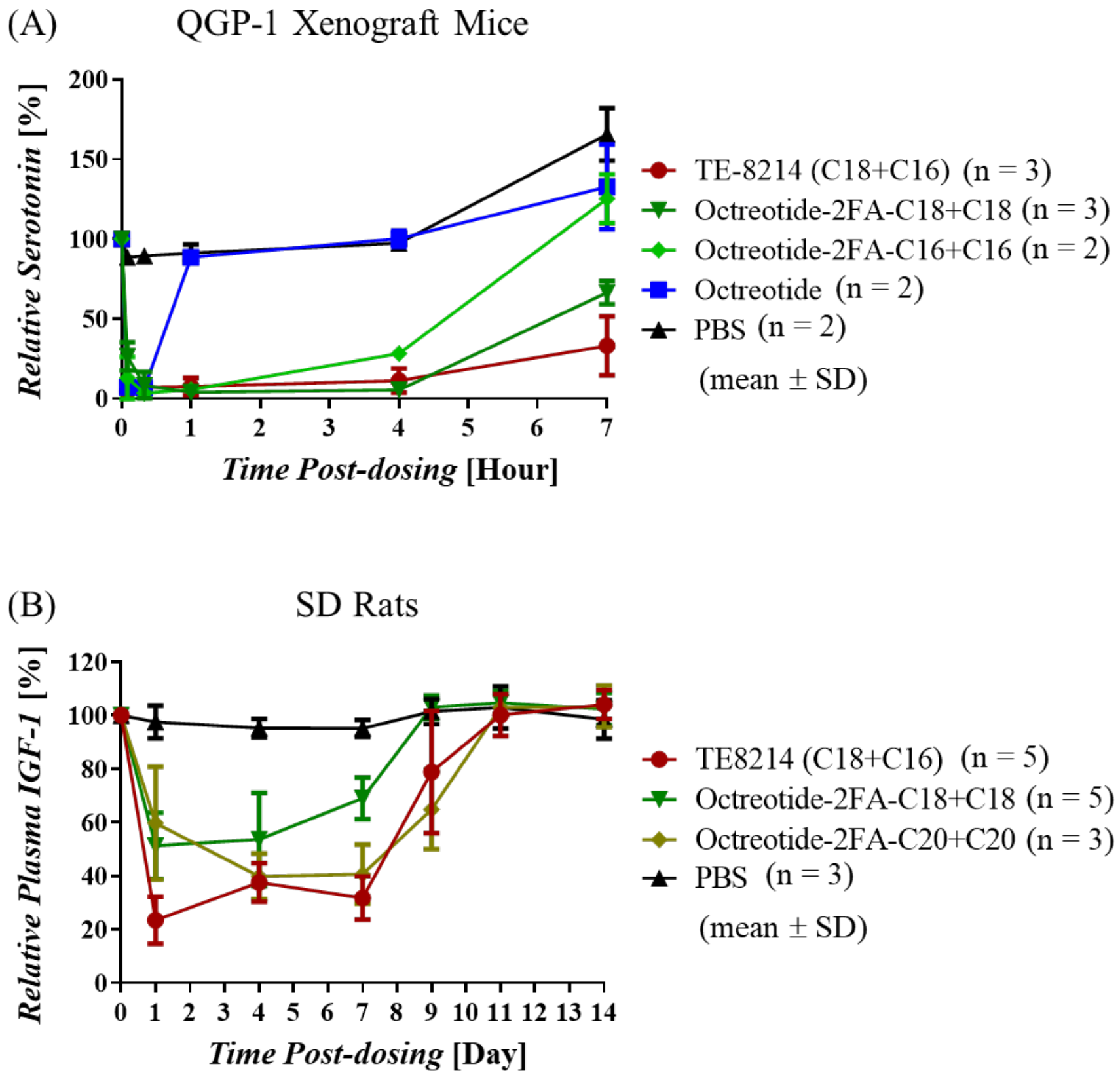

Supplementary Figure S3. TE-8214 Outlasts Equimolar Octreotide-2FA-C16+C16, Octreotide-2FA-C18+C18, and Octreotide-2FA-C20+C20 in Hormone Suppression. (A) Plasma serotonin (% pre-dose baseline) in NOD SCID mice with QGP-1 tumors after a single SC dose of TE-8214, Octreotide-2FA-C16+C16, Octreotide-2FA-C18+C18, (0.33 mg kg^{-1}), or phosphate-buffered saline (PBS). Data are mean ± SD; n = 2-3 per group. Octreotide-2FA-C16+C16 exhibited inferior maintenance of efficacy and less potent serotonin inhibition (as shown on Day 4 and Day 7) compared to Octreotide-2FA-C18+C18 and TE-8214. Therefore, Octreotide-2FA-C16+C16 was excluded from further analysis. (B) Plasma IGF-1 (% pre-dose baseline) in SD rats after a single SC dose of TE-8214, Octreotide-2FA-C18+C18, Octreotide-2FA-C20+C20 (330 μg kg^{-1}) or PBS. Data are mean ± SD; n = 3-5 per group. TE-8214 demonstrated the best sustained efficacy and IGF-1 suppression compared to Octreotide-2FA-C18+C18 and Octreotide-2FA-C20+C20.

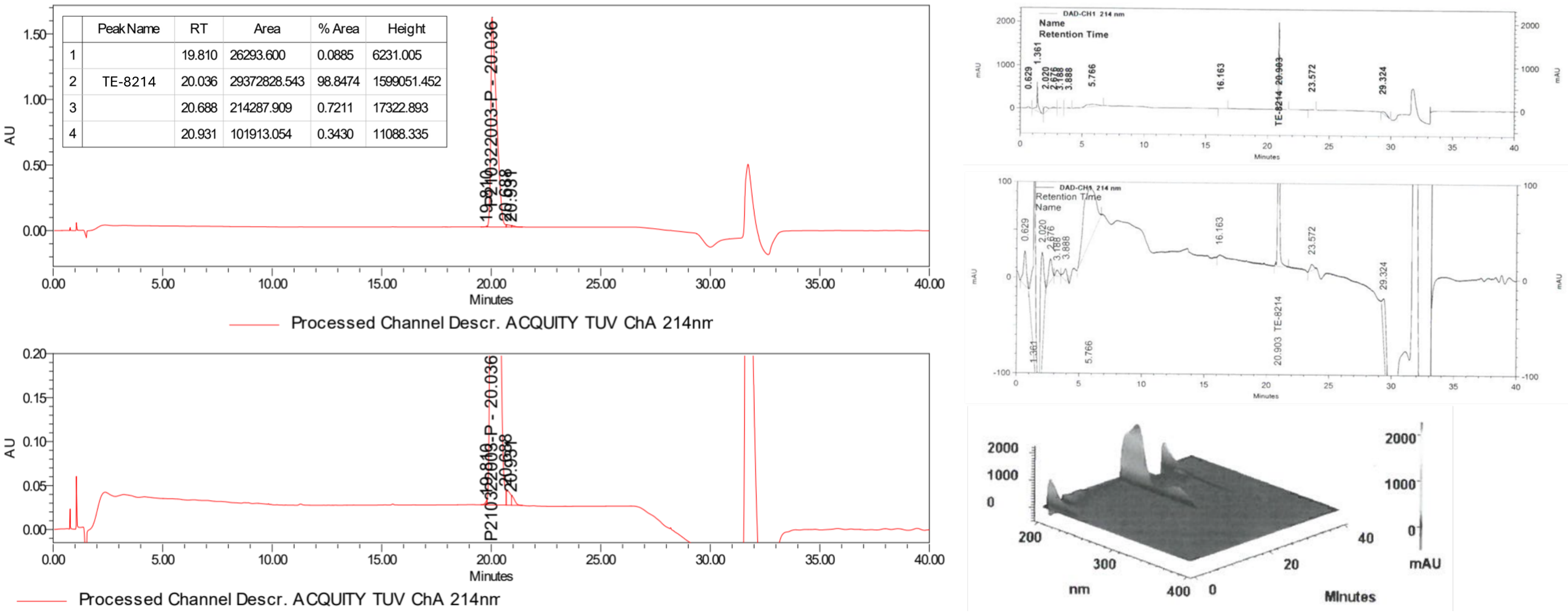

Supplementary Figure S4. Purity of TE-8214. (Left) The HPLC chromatogram displays the UV absorbance at 214 nm versus retention time. TE-8214 appears as a single dominant peak at its characteristic retention time. (Right) UPLC-UV chromatogram and UV spectrum of TE-8214.

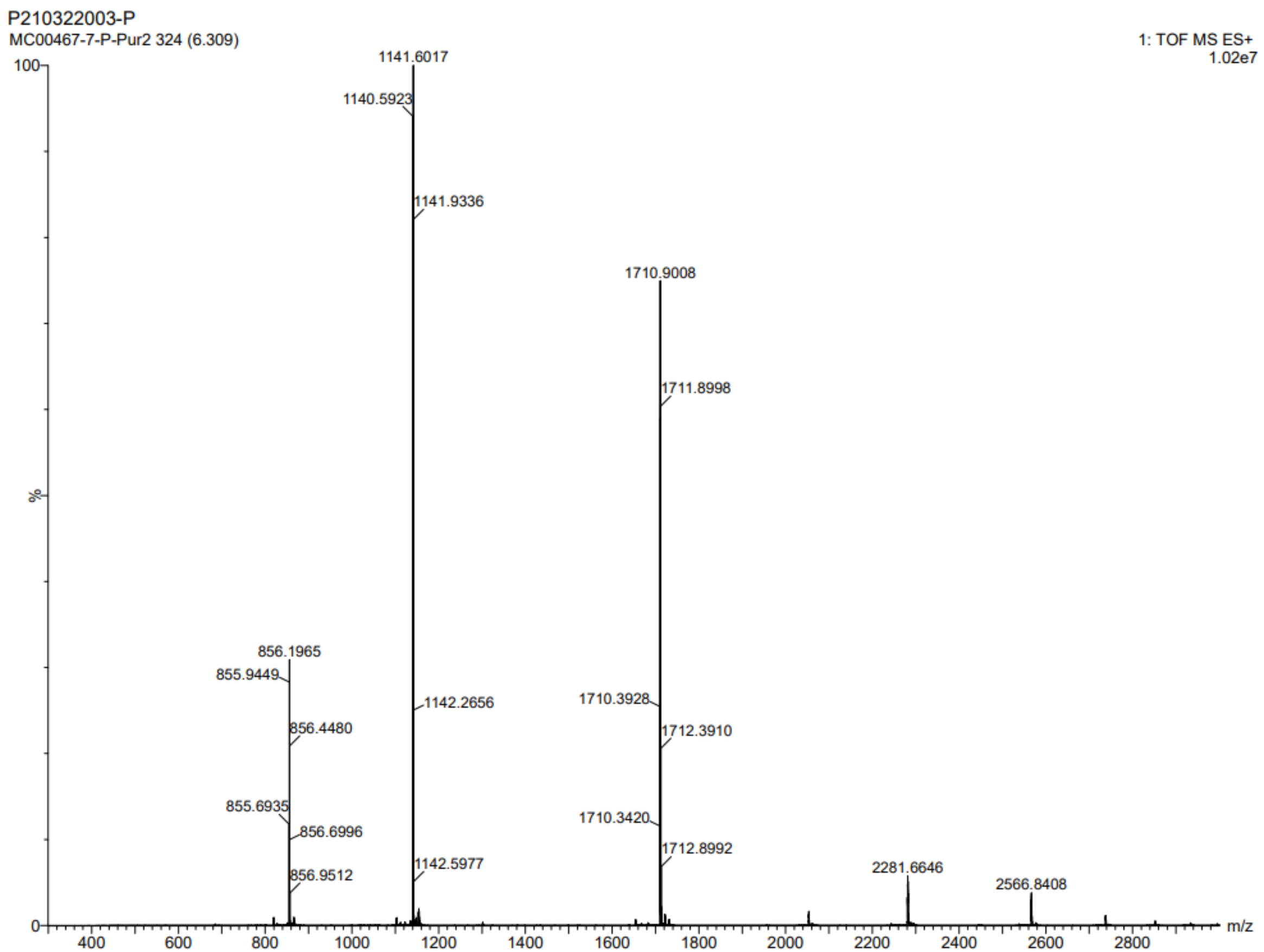

Supplementary Figure S5. Verification of TE-8214 using liquid chromatography-mass spectrometry (LC-MS). The LC-MS spectrum (mass spectrum) displays ion intensity versus mass-to-charge ratio (m/z). The deconvoluted spectrum shows a major peak corresponding to the molecular weight of TE-8214 (3418.71 Da), closely matching the theoretical value (3418.75 Da).

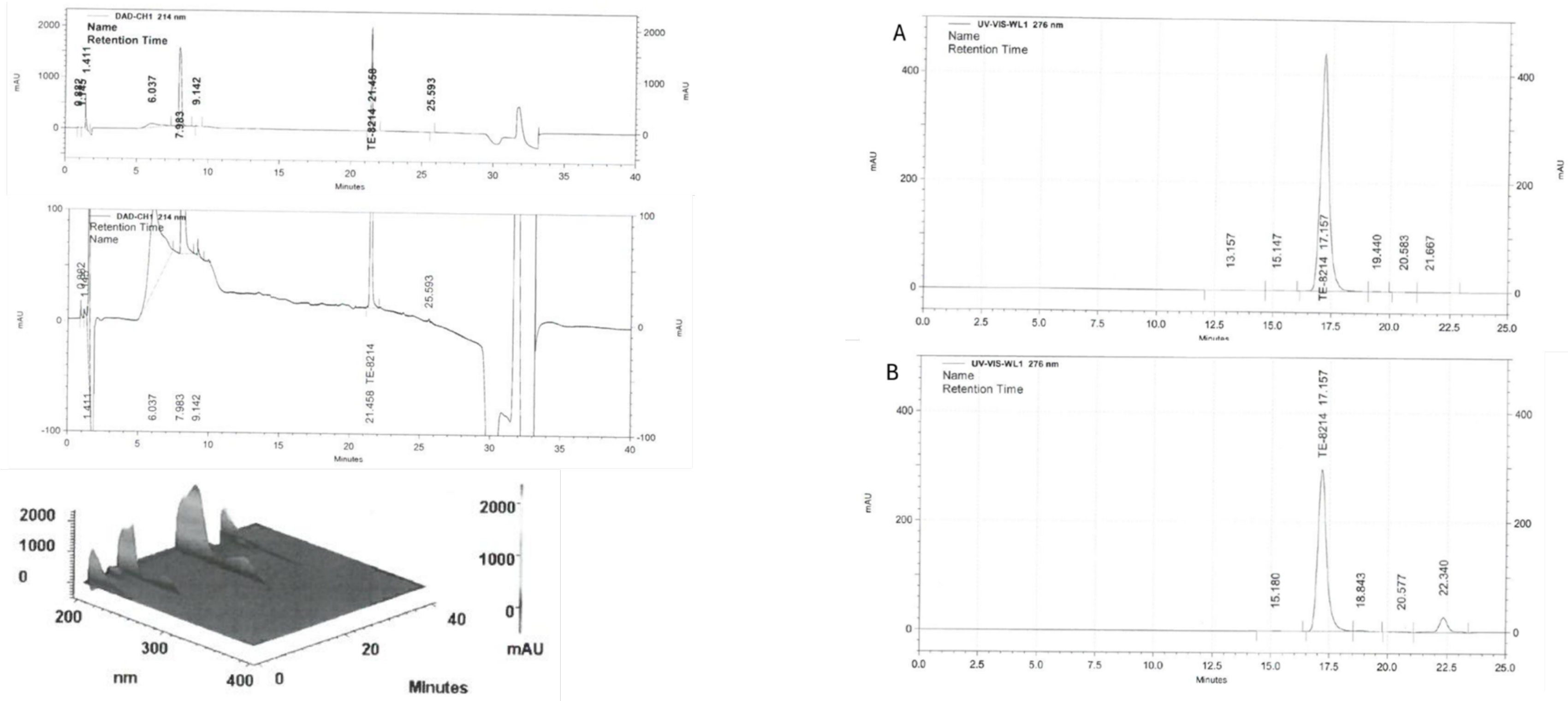

Supplementary Figure S6. TE-8214 Drug Product Retains Chemical Integrity and Purity After 24-Month Storage at 5 ± 3°C. (Left) UPLC-UV chromatogram and UV spectrum of formulated TE-8214 drug product after 24-month storage, confirming preserved chemical identity and purity relative to the reference standard (Supplementary Figure S4). (Right) SEC-HPLC chromatograms of (A) TE-8214 reference standard and (B) formulated TE-8214 drug product after 24-month storage, demonstrating the absence of high-molecular-weight aggregates or degradation species.

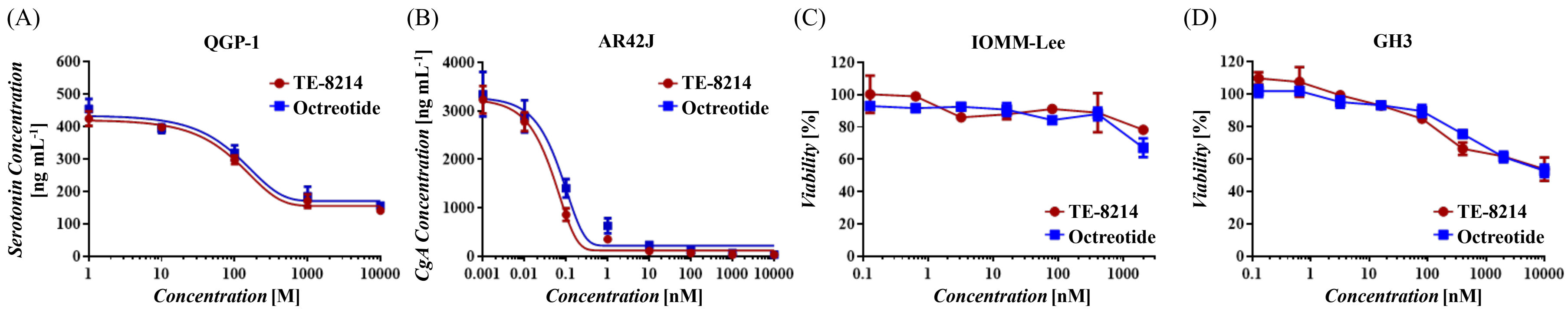

Supplementary Figure S7. TE-8214 Retains Octreotide-Like SSTR2-Mediated Potency. (A, B) Dose-dependent suppression of serotonin secretion from QGP-1 cells (A) and CgA expression in AR42J cells (B) after a 2-day incubation with TE-8214 or octreotide. (C, D) Dose-dependent reductions in cell viability in IOMM-Lee (C) and GH3 (D) cells after a 3-day incubation with TE-8214 or octreotide; data are mean ± SD; n = 3.

Table S1. Histopathology Changes of SD Rats at the End of Dosing Phase (Day 30) and Recovery Phase (Day 58)

Day 30

Sex		Male				Female			
mg/kg/Dose		0	2	6	18	0	2	6	18
No. of Animal Examined		10	10	10	10	10	10	10	10
**Injection Site**									
Inflammation, Mixed Cell, Subcutaneous									
	Minimal	2	3	4	6	8	5	4	6
	Mild	1	4	3	3	1	2	5	2
	Moderate	0	0	1	0	0	0	0	1
Degeneration/Regeneration, Muscle, Multifocal									
	Minimal	0	0	0	1	0	0	1	0
	Mild	0	1	0	0	0	0	1	0
Crust, Serocellular/Hyperplasia, Epidermis, Focal									
	Mild	0	0	0	1	0	0	0	0
Hemorrhage, Subcutaneous and/or Dermis									
	Minimal	0	4	3	4	5	1	1	4
	Mild	1	1	5	4	2	3	4	2
	Moderate	0	1	0	1	0	0	2	1

Day 58

Sex		Male				Female			
mg/kg/Dose		0	2	6	18	0	2	6	18
No. of animal Examined		5	5	5	5	5	5	5	5
**Injection Site**									
Inflammation, Mixed Cell, Subcutaneous									
	Minimal	3	0	1	1	3	1	4	3
Hemorrhage, Subcutaneous									
	Minimal	1	0	0	0	0	0	0	1

Table S2. Summary of Local Irritation Evaluation in SD rats

Score From Day 1 (Start Date) to Day 58 (End Date)		Gender	Male				Female			
		Identity	Vehicle	TE-8214	TE-8214	TE-8214	Vehicle	TE-8214	TE-8214	TE-8214
		Dose Level (mg/kg)	0	2	6	18	0	2	6	18
Dosing Period-From Day 1 (Start Date) to Day 30 (End Date)										
Edema	Score 0									
	Number of Animals Affected		15	15	15	15	15	15	15	15
	First to Last seen		1 - 30	1 - 30	1 - 30	1 - 30	1 - 30	1 - 30	1 - 30	1 - 30
Erythema	Score 0									
	Number of Animals Affected		15	15	15	15	15	15	15	15
	First to Last seen		1 - 30	1 - 30	1 - 30	1 - 30	1 - 30	1 - 30	1 - 30	1 - 30
Recovery Period-From Day 31 (Start Date) to Day 58 (End Date)										
Edema	Score 0									
	Number of Animals Affected		5	5	5	5	5	5	5	5
	First to Last seen		31 - 58	31 - 58	31 - 58	31 - 58	31 - 58	31 - 58	31 - 58	31 - 58
Erythema	Score 0									
	Number of Animals Affected		5	5	5	5	5	5	5	5
	First to Last seen		31 - 58	31 - 58	31 - 58	31 - 58	31 - 58	31 - 58	31 - 58	31 - 58

Table S3. Summary of Toxicokinetic Parameters in SD rats

Day	Dose Level (mg/kg)	Gender	T_{max} (h)		C_{max} (ng/mL)		$AUC_{(0-96)}$ (h·ng/mL)		Gender Difference Analysis[a]	Accumulation Analysis[b]
			Mean	SD	Mean	SD	Mean	SD		
1 (1st Dosing)	2	Male	24.00	0.00	6704	382	381455	13690	1.04	-
		Female	24.00	0.00	6593	462	367849	32609		
	6	Male	24.00	0.00	19442	3231	1125199	164246	0.94	-
		Female	24.00	0.00	20960	2151	1201265	115012		
	18	Male	30.00	12.00	48105	6590	3168210	332787	0.92	-
		Female	36.00	13.86	58216	10715	3439464	362423		
26 (8th Dosing)	2	Male	24.00	0.00	7760	897	520009	44496	0.93	1.36
		Female	18.00	6.93	9083	1321	556321	33982		1.52
	6	Male	24.00	0.00	24301	1670	1670206	170562	0.99	1.49
		Female	24.00	0.00	27165	2676	1691549	87547		1.42
	18	Male	42.00	12.00	68180	9820	5115842	377091	0.88	1.63
		Female	24.00	0.00	89024	11658	5790676	671242		1.69

SD: Standard deviation.
[a] The $AUC_{(0-96)}$ ratio between males and females.
[b] The $AUC_{(0-96)}$ ratio between Day 26 and Day 1.

Table S4. Summary of Toxicokinetic Parameters in Beagle Dogs

Day	Dose Level (mg/kg)	Gender	T_{max} (h)		C_{max} (ng/mL)		$AUC_{(0-168)}$ (h·ng/mL)		Gender Difference Analysis[a]	Accumulation Analysis[b]
			Mean	SD	Mean	SD	Mean	SD		
1 (1st Dosing)	0.8	Male	52.80	26.29	5168	424	691389	55623	1.03	-
		Female	38.40	13.15	5197	431	671301	71432		
	2.5	Male	48.00	0.00	16355	1527	2137131	205964	1.05	-
		Female	52.80	10.73	15420	1743	2040903	156661		
	7.0	Male	76.80	20.08	40241	5576	5488228	818354	0.94	-
		Female	57.60	13.15	45807	3830	5853592	730584		
22 (4th Dosing)	0.8	Male	38.40	13.15	9092	994	1227677	116094	1.02	1.78
		Female	43.20	10.73	9100	1033	1204162	130144		1.80
	2.5	Male	38.40	13.15	28756	2541	3829779	266581	1.02	1.80
		Female	33.60	13.15	29509	3059	3766724	372703		1.85
	7.0	Male	67.20	10.73	74661	10011	10350722	1057923	0.95	1.90
		Female	57.60	13.15	82001	10508	10882486	1263020		1.86

SD: Standard deviation.

[a] The $AUC_{(0-168)}$ ratio between males and females.

[b] The $AUC_{(0-168)}$ ratio between Day 22 and Day 1.

Table S5. Histopathology Changes of Beagle Dogs at the end of Dosing Phase (Day 30) and Recovery Phase (Day 58)

Day 30

Sex		Male				Female			
mg/kg/Dose		0	0.8	2.5	7.0	0	0.8	2.5	7.0
No. of animal Examined		3	3	3	3	3	3	3	3
**Injection Site**									
Hemorrhage, Subcutaneous									
	Minimal	0	2	1	1	1	2	1	2
	Mild	2	0	0	1	1	0	2	0
Inflammation, Subcutaneous, mixed cells									
	Minimal	2	1	1	1	0	1	1	1
	Mild	0	1	1	2	1	0	2	2
Infiltration, Mixed cell, dermis/subcutaneous									
	Minimal	0	1	0	0	0	0	0	0
**Liver**									
Hypertrophy, centrilobular, hepatocyte, diffuse									
	Minimal	0	0	0	0	0	0	0	1
Vacuolation, hepatocyte, multifocal									
	Minimal	0	0	0	1	0	0	0	0
**Spleen**									
Congestion, red pulp, multifocal/diffuse									
	Minimal	1	0	0	0	0	1	1	1
	Mild	0	0	0	1	0	0	0	1
	Marked	0	0	0	0	0	0	0	1
**Testes**									
Degeneration/atrophy, seminiferous tubules, unilateral, focal									
	Minimal	0	0	0	2	NA	NA	NA	NA

Sex		Male				Female			
mg/kg/Dose		0	0.8	2.5	7.0	0	0.8	2.5	7.0
No. of animal Examined		3	3	3	3	3	3	3	3
**Thymus**									
Decreased cellularity, Lymphocyte, Cortex, Diffuse									
	Minimal	0	2	0	1	0	1	0	3
	Mild	0	0	0	1	0	0	2	0
Lymphoid follicle, medulla, multifocal									
	Marked	0	0	0	1	0	0	0	0

Day 58

Sex		Male				Female			
mg/kg/Dose		0	0.8	2.5	7.0	0	0.8	2.5	7.0
No. of Animal Examined		2	2	2	2	2	2	2	2
**Injection Site**									
Hemorrhage, Subcutaneous									
	Minimal	0	1	0	0	1	1	0	0
	Mild	0	0	1	0	0	0	0	0
Inflammation, Subcutaneous, mixed cells									
	Minimal	0	1	1	0	0	1	0	0
**Liver**									
Hypertrophy, centrilobular, hepatocyte, diffuse									
	Minimal	0	0	0	0	0	1	0	0
Vacuolation, hepatocyte, multifocal									
	Minimal	0	0	1	1	0	1	0	0
**Spleen**									
Congestion, red pulp, multifocal/diffuse									

Sex		Male				Female			
mg/kg/Dose		0	0.8	2.5	7.0	0	0.8	2.5	7.0
No. of Animal Examined		2	2	2	2	2	2	2	2
	Minimal	0	0	1	0	0	0	0	1
	Mild	2	2	1	1	2	1	0	0
	Moderate	0	0	0	1	0	1	1	0
**Thymus**									
Decreased cellularity, Lymphocyte, Cortex, Diffuse									
	Minimal	0	0	0	2	0	0	0	0
	Mild	0	0	0	0	0	0	1	1

Table S6. Summary of Local Irritation Evaluation in Beagle Dogs

Score From Day 1 (Start Date) to Day 58 (End Date)		Gender	Male				Female			
		Identity	Vehicle	TE-8214	TE-8214	TE-8214	Vehicle	TE-8214	TE-8214	TE-8214
		Dose Level (mg/kg)	0	0.8	2.5	7.0	0	0.8	2.5	7.0
Dosing Period-From Day 1 (Start Date) to Day 30 (End Date)										
Edema	Score 0									
	Number of Animals Affected		5	5	5	5	5	5	5	5
	First to Last seen		1 - 30	1 - 30	1 - 30	1 - 30	1 - 30	1 - 30	1 - 30	1 - 30
Erythema	Score 0									
	Number of Animals Affected		5	5	5	5	5	5	5	5
	First to Last seen		1 - 30	1 - 30	1 - 30	1 - 30	1 - 30	1 - 30	1 - 30	1 - 30
Recovery Period-From Day 31 (Start Date) to Day 58 (End Date)										
Edema	Score 0									
	Number of Animals Affected		2	2	2	2	2	2	2	2
	First to Last seen		31 - 58	31 - 58	31 - 58	31 - 58	31 - 58	31 - 58	31 - 58	31 - 58
Erythema	Score 0									
	Number of Animals Affected		2	2	2	2	2	2	2	2
	First to Last seen		31 - 58	31 - 58	31 - 58	31 - 58	31 - 58	31 - 58	31 - 58	31 - 58

Table S7. Calculation of Safety Margin

Species	Animal NOAEL (mg/kg)	FDA allometric conversion factor	Human Equivalent Dose HED (mg/kg)	4 mg Human Dose Conversion (mg/kg)[a]	Safety Margin[b]
Rat	18.0	6.2	2.9	0.067	43.3-fold
Dog	2.5	1.8	1.4	0.067	20.9-fold

[a] Based on 60 kg human. [b]Safety Margin = HED/4 mg human dose conversion.

Supplementary Table S8. Adverse Events in the Phase 1 Study[a]

# of participants reporting	0.6 mg (N=6)	1.2 mg (N=6)	2.0 mg (N=6)	4.0 mg (N=6)	Placebo (N=8)
≥ 1 SAEs	0	0	0	0	0
≥ Grade 3 TEAE	0	0	0	0	0
≥ 1 TEAE	4	5	3	5	7

[a]Adverse events were coded using MedDRA (Medical Dictionary for Regulatory Activities) version 27.0; severity was graded using the National Cancer Institute Common Terminology Criteria for Adverse Events version 5.0, where grades 1-5 indicate mild, moderate, severe, life-threatening and death, respectively. Serious adverse events (SAEs) are defined by outcome criteria, such as death, life-threatening situations, hospitalization, or permanent disability, regardless of the event's grade. TEAEs are defined as adverse events occurring after first administration of study medication.

Supplementary Table S9. Summary of TEAEs by System Organ Class and Preferred Term (Safety Population) in the Phase 1 Study (TE-8214-001)[a]

System Organ Class<br>Preferred Term	TE-8214 (0.6 mg) (N=6) n (%) m	TE-8214 (1.2 mg) (N=6) n (%) m	TE-8214 (2.0 mg) (N=6) n (%) m	TE-8214 (4.0 mg) (N=6) n (%) m	Placebo (N=8) n (%) m	TE-8214 Overall (N=24) n (%) m
Participants with at least one TEAE	4 (66.7) 6	5 (83.3) 10	3 (50.0) 3	5 (83.3) 9	7 (87.5) 13	17 (70.8) 28
Injury, poisoning and procedural complications	2 (33.3) 3	2 (33.3) 5	1 (16.7) 1	2 (33.3) 2	4 (50.0) 6	7 (29.2) 11
Vascular access site pain	0	2 (33.3) 3	0	1 (16.7) 1	2 (25.0) 3	3 (12.5) 4
Vascular access site bruising	1 (16.7) 1	2 (33.3) 2	0	0	3 (37.5) 3	3 (12.5) 3
Injection site bruising	2 (33.3) 2	0	0	0	0	2 (8.3) 2
Injection site erythema	0	0	1 (16.7) 1	1 (16.7) 1	0	2 (8.3) 2
Gastrointestinal disorders	1 (16.7) 1	0	0	2 (33.3) 3	0	3 (12.5) 4
Abdominal pain	0	0	0	1 (16.7) 1	0	1 (4.2) 1
Diarrhea	0	0	0	1 (16.7) 1	0	1 (4.2) 1
Feces soft	0	0	0	1 (16.7) 1	0	1 (4.2) 1
Gastroenteritis	1 (16.7) 1	0	0	0	0	1 (4.2) 1
Nervous system disorders	1 (16.7) 1	1 (16.7) 1	0	1 (16.7) 2	1 (12.5) 1	3 (12.5) 4

System Organ Class<br>Preferred Term	TE-8214<br>(0.6 mg)<br>(N=6)<br>n (%) m	TE-8214<br>(1.2 mg)<br>(N=6)<br>n (%) m	TE-8214<br>(2.0 mg)<br>(N=6)<br>n (%) m	TE-8214<br>(4.0 mg)<br>(N=6)<br>n (%) m	Placebo<br>(N=8)<br>n (%) m	TE-8214<br>Overall<br>(N=24)<br>n (%) m
Headache	0	1 (16.7) 1	0	1 (16.7) 1	1 (12.5) 1	2 (8.3) 2
Dizziness	1 (16.7) 1	0	0	0	0	1 (4.2) 1
Vertigo	0	0	0	1 (16.7) 1	0	1 (4.2) 1
Investigations	0	2 (33.3) 2	0	0	1 (12.5) 1	2 (8.3) 2
Alanine aminotransferase abnormal	0	1 (16.7) 1	0	0	1 (12.5) 1	1 (4.2) 1
Alanine aminotransferase increased	0	1 (16.7) 1	0	0	0	1 (4.2) 1
Musculoskeletal and connective tissue disorders	1 (16.7) 1	0	0	1 (16.7) 1	1 (12.5) 1	2 (8.3) 2
Arthralgia	1 (16.7) 1	0	0	0	0	1 (4.2) 1
Muscle spasms	0	0	0	1 (16.7) 1	0	1 (4.2) 1
Back pain	0	0	0	0	1 (12.5) 1	0
Respiratory, thoracic and mediastinal disorders	0	0	1 (16.7) 1	1 (16.7) 1	0	2 (8.3) 2
Viral upper respiratory tract infection	0	0	1 (16.7) 1	1 (16.7) 1	0	2 (8.3) 2
Infections and infestations	0	0	1 (16.7) 1	0	0	1 (4.2) 1
COVID-19	0	0	1 (16.7) 1	0	0	1 (4.2) 1

System Organ Class<br>Preferred Term	TE-8214<br>(0.6 mg)<br>(N=6)<br>n (%) m	TE-8214<br>(1.2 mg)<br>(N=6)<br>n (%) m	TE-8214<br>(2.0 mg)<br>(N=6)<br>n (%) m	TE-8214<br>(4.0 mg)<br>(N=6)<br>n (%) m	Placebo<br>(N=8)<br>n (%) m	TE-8214<br>Overall<br>(N=24)<br>n (%) m
Renal and urinary disorders	0	1 (16.7) 1	0	0	0	1 (4.2) 1
Renal impairment	0	1 (16.7) 1	0	0	0	1 (4.2) 1
Vascular disorders	0	1 (16.7) 1	0	0	0	1 (4.2) 1
Presyncope	0	1 (16.7) 1	0	0	0	1 (4.2) 1
Blood and lymphatic system disorders	0	0	0	0	1 (12.5) 1	0
Neutropenia	0	0	0	0	1 (12.5) 1	0
Hepatobiliary disorders	0	0	0	0	1 (12.5) 1	0
Hypertransaminasemia	0	0	0	0	1 (12.5) 1	0
Skin and subcutaneous tissue disorders	0	0	0	0	2 (25.0) 2	0
Dermatitis contact	0	0	0	0	1 (12.5) 1	0
Pruritus	0	0	0	0	1 (12.5) 1	0

[a]Adverse events were coded using MedDRA (Medical Dictionary for Regulatory Activities) version 27.0, graded using the National Cancer Institute Common Terminology Criteria for Adverse Events version 5.0, where grades 1-5 indicate mild, moderate, severe, life-threatening and

death, respectively; TEAEs, (treatment-emergent adverse events) were defined as adverse events that occurred following the first administration of study medication. If a participant had multiple occurrences of a TEAE, the participant is presented only once in the Participant count (n) column for a given System Organ Class and Preferred Term. Occurrences are counted each time in the mentions (m) column.
Percentages were calculated based on the number of participants in the Safety Population in each treatment group (N).
